# Pyroptosis and mitochondrial function participated in miR-654-3p-protected against myocardial infarction

**DOI:** 10.1038/s41419-024-06786-4

**Published:** 2024-06-04

**Authors:** Chan Wu, Xiao-Cheng Zhang, Lan-Ruo Chen, Hui-Zhu Huang, Wei-Yin Wu, Yan Wang, Gang Li

**Affiliations:** Xiamen Key Laboratory of Cardiovascular Diseases, Xiamen Cardiovascular Hospital of Xiamen University, School of Medicine, Xiamen University, Xiamen, Fujian 361000 China

**Keywords:** Myocardial infarction, Translational research

## Abstract

Myocardial infarction (MI) is one of the leading causes of heart failure with highly complicated pathogeneses. miR-654-3p has been recognized as a pivotal regulator of controlling cell survival. However, the function of miR-654-3p in cardiomyocytes and MI has yet to be reported. This study aimed to identify the role of miR-654-3p in the regulation of myocardial infarction. To understand the contribution of miR-654-3p on heart function, we generated cardiac-specific knockdown and overexpression mice using AAV9 technology in MI injury. Mechanically, we combined cellular and molecular techniques, pharmaceutical treatment, RNA sequencing, and functional testing to elucidate the potential pathological mechanisms. We identified that mice subjected to MI decreased the expression of miR-654-3p in the border and infarcted area. Mice lacking miR-654-3p in the heart showed some inflammation infiltration and myocardial fibrosis, resulting in a mild cardiac injury. Furthermore, we found a deficiency of miR-654-3p in cardiomyocytes resulted in pyroptotic cell death but not other programmed cell death. Intriguingly, miR-654-3p deficiency aggravated MI-induced cardiac dysfunction, accompanied by higher myocardial fibrosis and cardiac enzymes and augmented pyroptosis activation. Cardiac elevating miR-654-3p prevented myocardial fibrosis and inflammation infiltration and decreased pyroptosis profile, thereby attenuating MI-induced cardiac damage. Using RNA sequence and molecular biological approaches, we found overexpression of miR-654-3p in the heart promoted the metabolic ability of the cardiomyocytes by promoting mitochondrial metabolism and mitochondrial respiration function. Our finding identified the character of miR-654-3p in protecting against MI damage by mediating pyroptosis and mitochondrial metabolism. These findings provide a new mechanism for miR-654-3p involvement in the pathogenesis of MI and reveal novel therapeutic targets.

**Figure:**
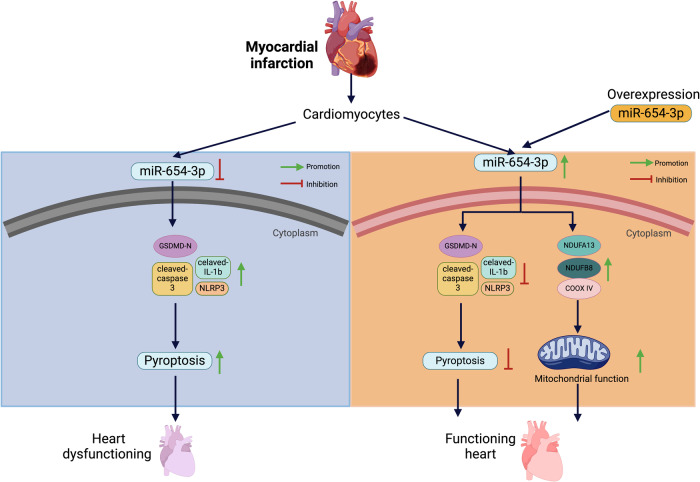
miR-654-3p expression was decreased after MI. Mice lacking miR-654-3p in the heart showed some inflammation infiltration and myocardial fibrosis, resulting in a mild cardiac injury. The deficiency of miR-654-3p in cardiomyocytes resulted in pyroptotic cell death. miR-654-3p deficiency aggravated MI-induced cardiac dysfunction, accompanied by higher myocardial fibrosis and cardiac enzymes and augmented pyroptosis activation. Overexpression of miR-654-3p prevented myocardial fibrosis and inflammation infiltration and decreased pyroptosis profile, thereby attenuating MI-induced cardiac damage. Overexpression of miR-654-3p in the heart promoted the metabolic ability of the cardiomyocytes by promoting mitochondrial metabolism and mitochondrial respiration function.

miR-654-3p expression was decreased after MI. Mice lacking miR-654-3p in the heart showed some inflammation infiltration and myocardial fibrosis, resulting in a mild cardiac injury. The deficiency of miR-654-3p in cardiomyocytes resulted in pyroptotic cell death. miR-654-3p deficiency aggravated MI-induced cardiac dysfunction, accompanied by higher myocardial fibrosis and cardiac enzymes and augmented pyroptosis activation. Overexpression of miR-654-3p prevented myocardial fibrosis and inflammation infiltration and decreased pyroptosis profile, thereby attenuating MI-induced cardiac damage. Overexpression of miR-654-3p in the heart promoted the metabolic ability of the cardiomyocytes by promoting mitochondrial metabolism and mitochondrial respiration function.

## Introduction

Myocardial infarction (MI) is a critical heart disease that affects millions of patients worldwide [1]. MI is characterized by decreased coronary blood flow, which causes insufficient oxygen supply to cardiac tissue [2]. MI usually results from an abrupt coronary occlusion that leads to ischemia and necrosis of the myocardium in the corresponding perfusion area. Prolonged ischemia can develop into heart failure (HF) and impaired left ventricular pump function, eventually resulting in sudden cardiac death [3]. Intense inflammatory responses have been observed during MI, recognized as major catastrophic events accounting for excessive injury and dysregulation of ventricular remodeling [1, 4]. Cardiac ischemia also leads to mitochondrial malfunction, activation of the ischemic cascade, and cell death [5]. Treatment of the disease includes early intervention by oxygen supplementation, use of anticoagulants, and immediate reperfusion [2, 5, 6].

There is a lot of myocardial cell death post-acute MI [7]. Many forms of programmed cell death (PCD), including apoptosis, necroptosis, ferroptosis, and pyroptosis, play separate and critical roles during the pathological progression of MI [8–13]. Inflammasome activation and pyroptotic cell death have been identified in acute MI [10, 14, 15]. Inflammation plays different roles in developing cardiac remodeling following the onset of myocardial ischemia, and the population of inflammatory cells changes dynamically with the progression of myocardial ischemia [16]. These alterations in inflammatory patterns may have protective or detrimental effects on cardiac function during chronic ischemia [17]. Pyroptosis is an inflammation-mediated cell death due to the priming and activating of the inflammasome, which subsequently increases the permeability of the plasma membrane and releases inflammatory cytokines [18]. It is vital to investigate the underlying mechanism and prevention approaches of MI-induced pyroptosis to help improve the understanding of the disease and potentially indicate new therapeutic targets.

In cancer cells, miR-654 contributes to the activation and proliferation in hepatic stellate cells [19], suppresses tumor growth in non-small cell lung cancer [20], prevents migration and proliferation in sinonasal squamous cell carcinoma [21], and promotes gastric cancer progression [22]. miR-654-3p is proven as a predictor for the prognosis of hepatocellular carcinoma and inhibits the proliferation, migration, and invasion of cancer cells [23]. MiR-654-3p has also been reported to regulate gastric cancer progression and glycolysis [24]. In vascular, miR-654-3p has also been reported to regulate migration and proliferation in vascular smooth muscle cells [25]. However, the role of the miR-654-3p on cardiomyocytes, cardiac function, and MI damage was poorly reported.

Here, we first explored the expression of miR-654-3p in MI mice. Next, we determined the role of miR-654-3p deficiency on cardiac function and further examined the potential mechanism in cardiomyocytes. Additionally, we examined the effect of miR-654-3p deficiency on MI-induced heart damage and investigated the therapeutic potential of overexpression of miR-654-3p on MI injury. The deep mechanism of miR-654-3p was also investigated using pharmacological treatments, RNA-sequence, and the seahorse mitochondrial function detection in neonatal rat cardiomyocytes (NRCMs) and myocardium. Through this study, we will identify a novel molecule for its potential therapeutic role in MI treatment.

## Results

### miR-654-3p is downregulated in the infarct area of the heart

**Fig. 1:**
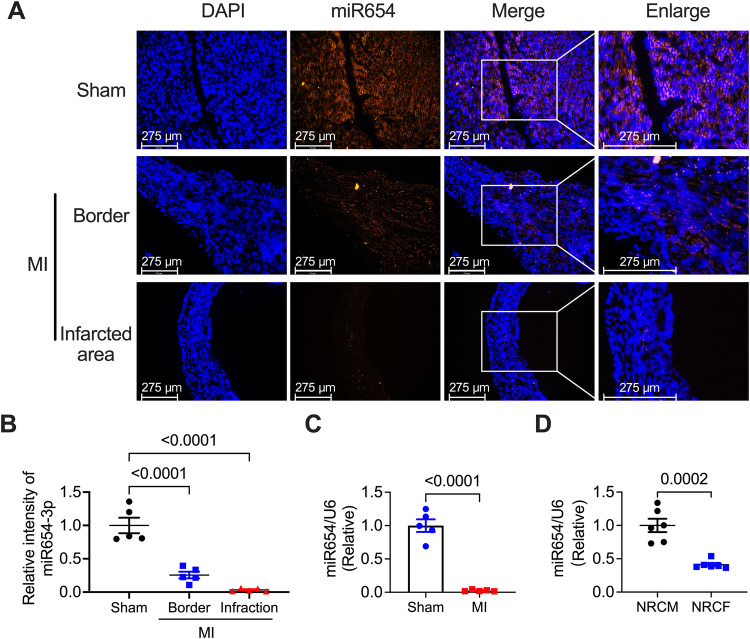
MI reduced miR-654-3p expression in the infarcted area of mice. **A** RNA-fluorescence in situ hybridization images and analysis (**B**) of miR-654-3p levels in the sham hearts and the border and infarcted area of the heart of MI mice (n = 5 in each group). **C** Quantitative real-time polymerase chain reaction (qRT-PCR) analysis of miR-654-3p expression level in the hearts of the sham and MI mice (n = 5 in each group). **D** qRT-PCR analysis of miR-654-3p expression level in the NRCMs and NRCFs isolated from newborn Sprague Dawley rats (*n* = 6 in each group). Data were presented as mean ± standard error. Statistical significance was assessed by one-way ANOVA (**B**, **D**) or T-tests **(C)**. MI Myocardial infarction; qRT-PCR Quantitative real-time polymerase chain reaction; NRCMs Neonatal rat cardiomyocyte; NRCF Neonatal rat cardiac fibroblast.

We constructed a myocardial infarction model in mice to determine the expression of miR-654-3p in the ischemic heart. We first applied RNA-fluorescence in situ hybridization (RNA-FISH) and found a reduced expression in the border of the infarcted area and a significant decrease in the infarcted area (Fig. 1A and B). We then validated it using quantitative Polymerase Chain Reaction (qPCR), which showed a consistent decrease in the expression of miR-654-3p (Fig. 1C). Furthermore, the expression of miR-654-3p was dominantly expressed in NRCMs but not the neonatal rat cardiac fibroblasts (NRCFs) (Fig. 1D). In addition, we detected the miR-654-3p in different organs and found that miR-654-3p was predominantly expressed in the spleen, lungs, and hearts, compared to the liver and kidney (Supplementary Fig. 1). These results indicated that miR-654-3p may participate in the pathogenesis of MI.

### Cardiac-specific knockdown of miR-654-3p reduced LV function and promoted inflammation infiltration and myocardial fibrosis

**Fig. 2:**
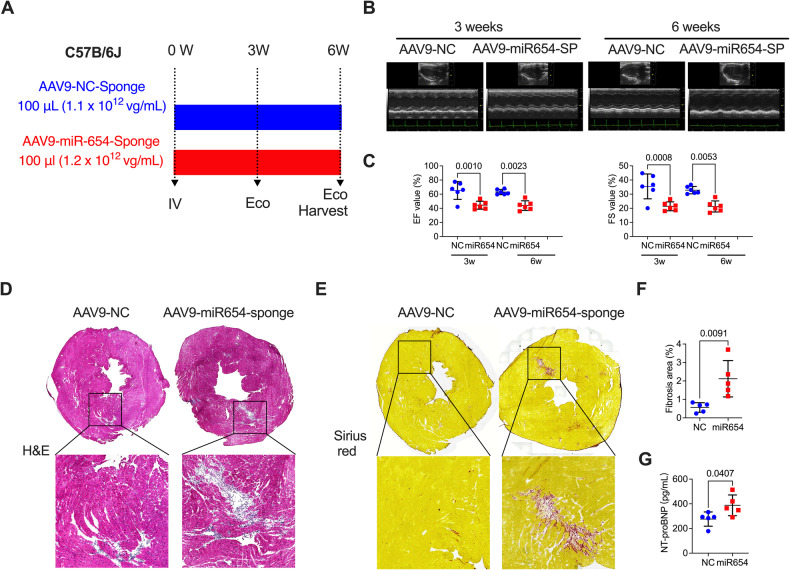
MiR-654-3p KO mice developed a mild cardiac injury. **A** Experimental timeline and design of cardia detection in AAV9-control (NC) and AAV9-miR-654-3p-sponge (SP)-infected C57B/6J mice. **B** Representative echocardiography images of AAV9-NC- or AAV9-miR-654-3p-SP-infected WT mice 3 and 6 weeks after infection. **C** Echocardiographic analysis of LV ejection fraction (LVEF) and LV fractional shortening (LVFS) in AAV9-NC or AAV9-miR-654-3p-SP infected mice at 3 or 6 weeks (*n* = 6 in each group). **D** Hematoxylin and eosin-stained heart of AAV9-NC or AAV9-miR-654-3p-SP infected mice (scale bar = 1 mm). **E** Representative Sirius red-stained heart and **F** analysis of the myocardial fibrosis in AAV9-NC- and AAV9-miR-654-3p-SP-infected mice (scale bar = 1 mm). **G** NT-proBNP levels in the serum of AAV9-NC- and AAV9-miR-654-3p-SP-infected mice (*n* = 5 in each group). Data were presented as mean ± standard error. Statistical significance was assessed by T-tests (**C**, **F**, and **G**). AAV Adeno-associated virus, NC Negative control, SP Sponge, LVEF Left ventricular ejection fraction, LVFS Left ventricular fractional shortening, H&E Hematoxylin and eosin, NT-proBNP N-terminal pro-brain Natriuretic Peptide.

To examine the influence of knockdown miR-654-3p on heart function, we constructed a miR-654-3p knockdown mouse model by infecting the heart with AAV9 (Fig. 2A). After infection for 3 weeks, the infection efficiency of AAV9-control-cTNT-Luc (AAV9-control) or AAV9-cTNT-Luc-miR-654-3p sponge (AAV9-miR-654-3p sponge) was captured by fluorescence microscopy, which showed a significant expression of luciferase, indicating a significant infection of the AAV9 in the heart (Supplementary Fig. 2A). The miR-654-3p expression in the heart was validated using qPCR, which exhibited a considerable reduction of miR-654-3p in AAV9-miR-654-3p sponge infection when compared with the AAV9-control (Supplementary Fig. 2B). We evaluated cardiac function 3- and 6 weeks after infection using echocardiography (ECG). The Left ventricular dysfunction was induced at 3 and 6 weeks in AAV9-miR-654-3p sponge-infected mice owing to the reduced left ventricular ejection fraction (LVEF) and left ventricular fractional shortening (LVFS) (Fig. 2B, C). The pathological staining, including picrosirius red and Hematoxylin and Eosin (H&E) staining, was employed to detect heart structure affected by the AAV9-miR-654-3p sponge. H&E staining showed a slightly promoted inflammation infiltration in the heart (Fig. 2D). Myocardial fibrosis was also shown in AAV9-miR-654-3p sponge-infected mice (Fig. 2E, F). We detected the N-terminal pro-brain natriuretic peptide (NT-proBNP), a cardiac hormone for the diagnosis of patients with acute or chronic heart failure, in the blood of the AAV9-control and AAV9-miR-654-3p sponge-infected mice, which showed a slight promotion in the AAV9-miR-654-3p group (Fig. 2G). These data indicated that miR-654-3p deletion induced a functional injury in the heart.

### Knockdown (KD) of miR-654-3p promoted NRCM death but not via apoptosis-dependent manner

**Fig. 3:**
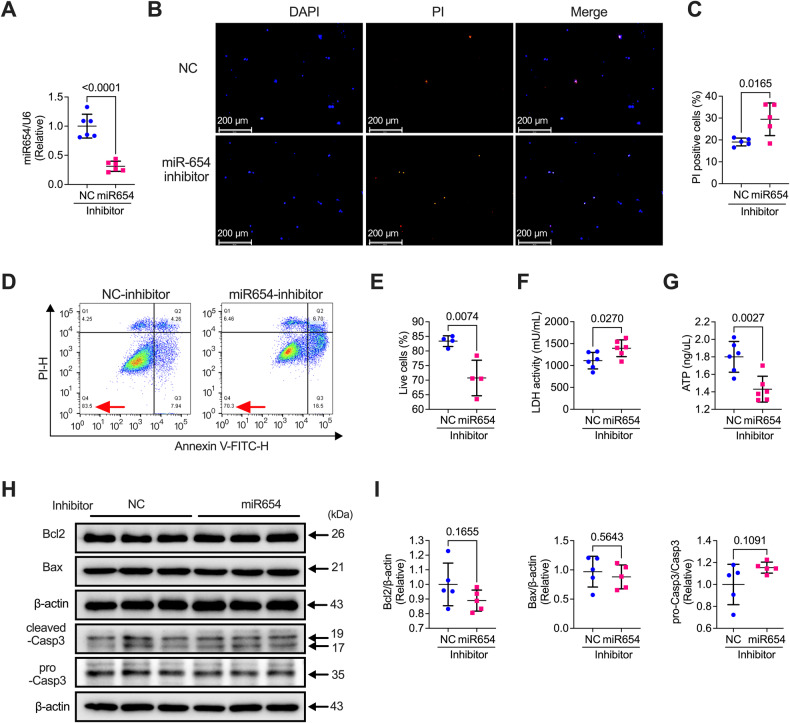
miR-654-3p deficiency reduced NRCMs viability not via regulating apoptosis. **A** miR-654-3p expression in Control (NC)- or miR-654-3p inhibitor-transfected NRCMs detected by qRT-PCR (*n* = 6 in each group). **B** Representative images of PI staining and **C** analysis of PI-positive NRCMs transfected with NC or miR-654-3p inhibitor (*n* = 5 in each group, scale bar = 200 μm). **D** Representative flow cytometry images and **E** analysis of cell apoptosis assay in NRCMs transfected with NC or miR-654-3p inhibitors (*n* = 6 in both groups). **F** LDH activity in NC- or miR-654-3p inhibitor-transfected NRCMs (*n* = 6 in both groups). **G** ATP production in control- or miR-654-3p inhibitor-transfected NRCMs (*n* = 6 in both groups). **H** Representative western blotting images and **I** quantitative analysis of apoptosis-related proteins in NRCMs transfected with control or miR-654-3p inhibitor (*n* = 5 in each group). Data were presented as mean ± standard error. Statistical significance was assessed by T-tests (**A**, **C**, **E**, **F**, **G**, and **I**). NC Negative control, NRCM Neonatal rat cardiomyocyte, qRT-PCR Quantitative real-time polymerase chain reaction, PI Propidium iodide, LDH Lactatedehydrogenase, ATP Adenosine triphosphate.

We next examined how the knockdown of miR-654-3p affected the function of the cardiomyocytes by transfection with the miR-654-3p inhibitor in NRCMs. The expression of miR-654-3p was decreased in miR-654-3p inhibitor-transfected NRCMs (Fig. 3A). PI staining can reflect cell death; thus, we employed PI staining, which showed the PI-positive cells were increased after transfection with the miR-654-3p inhibitor (Fig. 3B, C). Flow cytometry detection using annexin V and propidium iodide (PI) staining demonstrated more death cells in miR-654-3p inhibitor-transfected cells than in the control (Fig. 3D, E). Additionally, the lactate dehydrogenase (LDH) activity was increased (Fig. 3F) and ATP production was decreased (Fig. 3G) in miR-654-3p inhibitor-transfected cells, implying cell death and damage occurred. Moreover, we examined the apoptosis-related protein in control inhibitor or miR-654-3p inhibitor-transfected NRCMs, which showed no significant differences in them (Fig. 3H, I). These data indicated that miR-654-3p induced NRCM death but not via apoptosis.

### Inhibition of miR-654-3p promoted pyroptosis in NRCMs

**Fig. 4:**
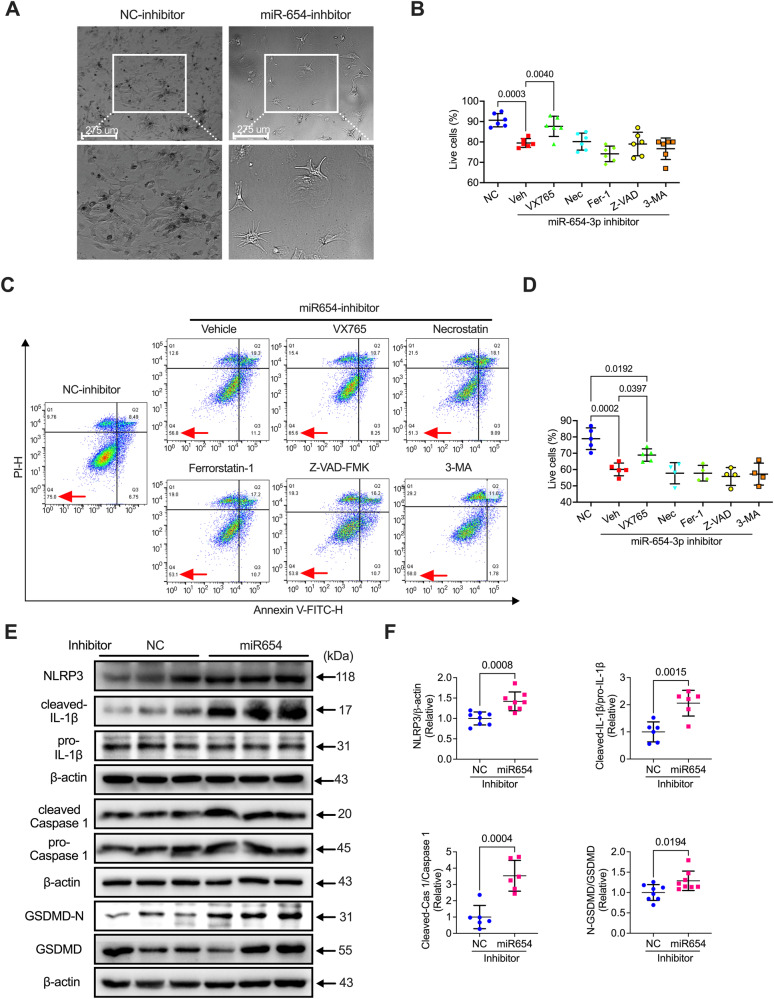
MiR-654-3p deficiency promotes pyroptosis in NRCMs. **A** Phase contrast images of NC- or miR-654-3p inhibitor-transfected NRCMs. **B** Cell viability in NC- or miR-654-3p inhibitor-transfected NRCMs treated with vehicle (Veh), VX765, Necrostatin (Nec), Ferrostatin-1 (Fer-1), Z-VAD-FMK (Z-VAD), and 3-methyladenine (3-MA) (*n* = 6 in each group). **C** Representative flow cytometry images and **D** analysis of cell apoptosis assay in NRCMs transfected with NC or miR-654-3p inhibitor (*n* = 5 in both groups). **E** Representative images of western blotting assay and **F** analysis of relative expression of pyroptosis-related proteins in NRCMs transfected with NC or miR-654-3p inhibitor (*n* = 6 or 8 in each group). Data were presented as mean ± standard error. Statistical significance was assessed by one-way ANOVA (**B**, **D**) and T-tests (**F**). NC Negative control, NRCM Neonatal rat cardiomyocyte, Veh vehicle, Nec Necrostatin, Fer-1 Ferrostatin-1, Z-VAD Z-VAD-FMK, 3-MA 3-methyladenine.

We further assessed how the miR-654-3p inhibition induced cell death. The cell morphology was observed in control inhibitor and miR-654-3p inhibitor transfected NRCMs. The cells displayed a deceased count and expanded size after transfection with miR-654-3p inhibitor (Fig. 4A). Further, we applied a series of inhibitors of programmed cell death, including pyroptosis inhibitor VX765, necroptosis inhibitor Necrostatin, ferroptosis inhibitor Ferrostatin-1, apoptosis inhibitor Z-VAD-FMK, and autophagy inhibitor 3-methyladenine (3-MA). In the cell viability assay, we found transfection with the miR-654-3p inhibitor induced a significantly decreased lived cells, which could be attenuated by pyroptosis inhibitor VX765, but not other inhibitors such as Necrostatin, Ferrostatin-1, Z-VAD-FMK, and 3-MA (Fig. 4B). Flow cytometry detection confirmed this effect, which showed that the death cells were significantly reduced in treating VX765 but not others (Fig. 4C and D). These results implied pyroptosis may participate in miR-654-3p-induced cell death. Moreover, we validated the hypothesis by measuring the pyroptosis-related protein. The result presented that cleaved interleukin 1 Beta (IL-1β), pro-IL-1β, cleaved Caspase 1, NLRP3, and N-terminal GSDMD were significantly promoted by transfection with the miR-654-3p inhibitor (Fig. 4E and F). These data indicated that miR-654-3p deficiency promoted pyroptosis in NRCMs.

### miR-654-3p deficiency aggravated MI injury

**Fig. 5:**
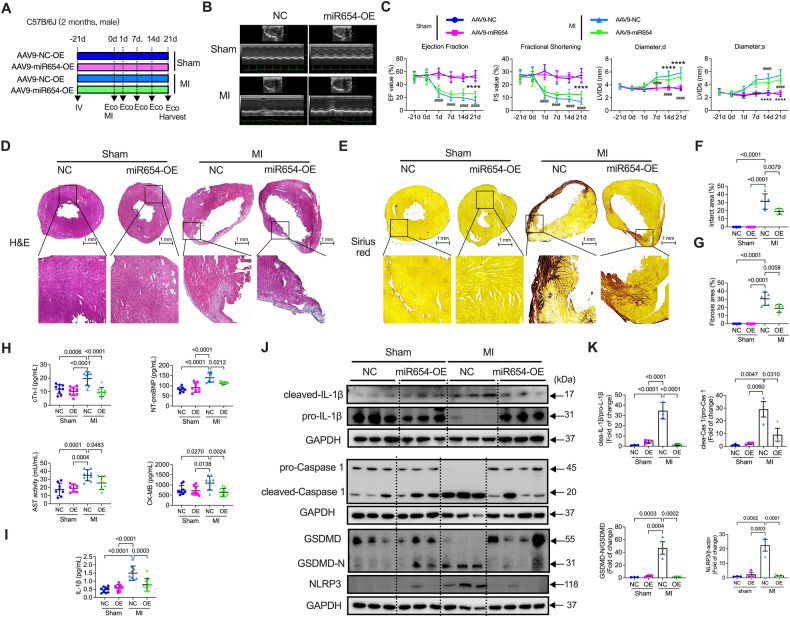
MiR-654-3p deficiency aggravated MI-induced cardiac dysfunction. **A** The experimental timeline of the MI model in AAV9-control (NC) and AAV9-miR-654-3p sponge (SP)-infected C57B/6J mice. **B** Representative M-mode echocardiographic images of sham or MI-induced mice treatment with AAV9-NC and AAV9-miR-654-3p-SP 1 and 21 days after surgery. **C** Echocardiography analysis of left ventricular (LV) ejection fraction (LVEF) and LV fractional shortening (LVFS) of sham or MI-induced mice treatment with AAV9-NC and AAV9-miR-654-SP 1 day (*n* = 8 in sham AAV9-NC, *n* = 10 in sham AAV9-miR-654-3p-SP, *n* = 12 in MI AAV9-NC, *n* = 12 in MI AAV9-miR-654-3p-SP) or **D** 21 days (*n* = 8 in sham AAV9-NC, *n* = 10 in sham AAV9-miR-654-3p-SP, *n* = 10 in MI AAV9-NC, *n* = 8 in MI AAV9-miR-654-3p-SP) after surgery. **E** Representative Hematoxylin and eosin (H&E) staining images and **F** quantification of infarct size in sham or MI mice treatment with AAV9-NC and AAV9-miR-654-3p-SP (*n* = 5 per group, scale bar = 1 mm). **G** Representative in Sirius red staining images and **H** quantification of the myocardial fibrotic area in sham or MI mice treatment with AAV9-NC and AAV9-miR-654-3p-SP (*n* = 5 per group; scale bar = 1 mm). **I** The serum concentration of cTn-I and NT-proBNP of AAV9-NC- and AAV9-miR-654-3p-SP-infected mice subjected to sham or MI (n = 6 or 7 in each group). **J** ELISA assay detected IL-1β and IL-18 in the serum of AAV9-NC- and AAV9-miR-654-3p-SP-infected mice subjected to sham or MI (*n* = 5 or 6 in each group). **K** Representative western blotting images and **L** quantitative analysis of pyroptosis-related proteins in the heart of AAV9-NC- or AAV9-miR-654-3p-SP-infected mice subjected to sham or MI (*n* = 3 in each group). Data were presented as mean ± standard error. Statistical significance was assessed by one-way ANOVA. MI Myocardial infarction; NC Negative control; SP Sponge; LVEF Left ventricular ejection fraction; LVFS Left ventricular fraction shortening; H&E Hematoxylin and eosin; cTn-I Troponin I Type 3, Cardiac; NT-proBNP N-terminal pro-brain Natriuretic Peptide; IL-1β Interleukin 1β; IL-18 Interleukin 18.

To further test whether miR-654-3p deficiency acts on MI injury, we constructed an MI model in AAV9-control and AAV9-miR-654-3p sponge-infected mice. Figure 5A shows the experimental design and timing of cardiac function measurements in the MI mouse model. MI impaired cardiac function by decreasing LVEF and LVFS at 1 and 21 days, whereas SP showed more decrease at 21 days after MI (Fig. 5B–D). Additionally, SP augmented the MI-induced infarction area (Fig. 5E–F) and myocardial fibrosis post-MI (Fig. 5G, H). Besides, we detected the myocardial enzyme Cardiac troponin I (cTNI) and heart failure marker NT-proBNP to verify the AAV9-miR-654-3p sponge-induced injury post-MI, which exhibited a significant promotion in the AAV9-miR-654-3p sponge group compared with the AAV9-control group (Fig. 5I). The levels of pyroptotic cytokines, IL-1β and IL-18, were considerably promoted in the AAV9-miR-654-3p sponge group post-MI (Fig. 5J). Further, we validated the expression of pyroptosis-related proteins in the heart. The results displayed that cleaved IL-1β, cleaved Caspase 1, GSDMD-N-terminal, and NLRP3 were promoted in the AAV9-control-infected heart of MI mice, and a significantly higher expression of them in the AAV9-miR-654-3p sponge-transfected MI mice (Fig. 5K, L). These data indicate that miR-654-3p deletion results in the aggravation of MI injury by promoting pyroptosis.

### Cardiac-specific overexpression of miR-654-3p alleviated myocardial injury post-MI

**Fig. 6:**
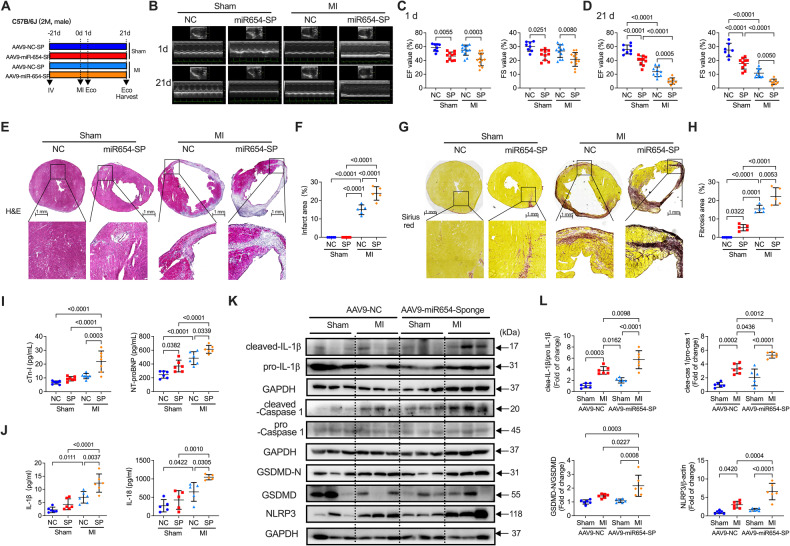
Cardiac-specific overexpression miR-654-3p attenuated MI-induced cardiac damage. **A** The experimental design of the MI model in AAV9-control (NC)- and AAV9-miR-654-3p-overexpression (OE)-infected C57B/6J mice. **B** Representative M-mode echocardiographic images of sham or MI-induced mice treatment with AAV9-NC and AAV9-miR-654-3p-OE. **C** Echocardiography analysis of LV ejection fraction and LV fractional shortening of sham or MI-induced mice treatment with AAV9-NC and AAV9-miR-654-3p-OE (n = 9-11 in different group) at -21, 0, 1, 7, 14, and 21 days after surgery. **D** Representative Hematoxylin and eosin (H&E) staining images and **F** quantification of infarct area in sham- or MI-induced mice treatment with AAV9-NC and AAV9-miR-654-3p-OE (*n* = 5 in each group, scale bar = 1 mm). **E** Representative Sirius red staining images and **G** quantification of the fibrotic area in sham- or MI-induced mice treatment with AAV9-NC or AAV9-miR-654-3p-OE (*n* = 5 in each group; scale bar = 1 mm). **H** The concentration of cTn-I and NT-proBNP, CK-MB, and AST in the serum of AAV9-NC- and AAV9-miR-654-3p-OE-infected mice subjected to sham or MI (*n* = 7 in each group). **I** ELISA assay detected IL-1β in the serum of AAV9-NC- or AAV9-miR-654-3p-OE-infected mice subjected to sham or MI (*n* = 8 or 9 in different group). **J** Representative western blotting images and **K** quantitative analysis of pyroptosis-related proteins in the heart of AAV9-NC- or AAV9-miR-654-3p-OE-infected mice subjected to sham or MI (*n* = 3 or 4 in different group). Data were presented as mean ± standard error. Statistical significance was assessed by one-way ANOVA. NC Negative control, OE overexpression, MI Myocardial infarction, LVEF Left ventricular ejection fraction, LVFS Left ventricular fraction shortening, H&E Hematoxylin and eosin, cTn-I Troponin I Type 3, Cardiac, NT-proBNP N-terminal pro-brain Natriuretic Peptide, CK-MB Creatine Kinase MB Isoenzyme, AST Aspartate Aminotransferase, IL-1β Interleukin 1β.

To investigate whether miR-654-3p overexpression has a therapeutic effect on MI-induced heart dysfunction, we injected AAV9-cTNT-mmu-mir-654-3p-LUC (AAV9-miR-654-3p-OE) 3 weeks before MI surgery, which specifically promoted the expression of miR-654-3p in the heart. By detecting the luciferase segment in AAV9 using an in vivo imaging system, the AAV9 particles were mainly expressed in the heart (Supplementary Fig. 3A). The expression of miR-654-3p in the hearts was validated by qPCR, which exhibited a significant promotion of miR-654-3p approximately 6-fold in the AAV9-miR-654-3p-OE-infected mice compared to AAV9-control-cTNT-Luc (AAV9-control) (Supplementary Fig. 3B). The experimental design is shown in Fig. 6A. ECG revealed that the MI-decreased LVEF and LVFS were not affected by AAV9-control but were significantly restored by AAV9-miR-654-3p-OE at 21 days post-MI (Fig. 6B, C). H&E and Sirius red staining found that miR-654-3p overexpression reduced myocardial infarct area (Fig. 6D, F) and myocardial fibrosis (Fig. 6E, G). Additionally, MI-increased levels of NT-proBNP, cTn-I, aspartate aminotransferase (AST), and creatine kinase MB Isoenzyme (CK-MB) in the serum were also reversed by overexpression of miR-654-3p (Fig. 6H) and the level of IL-1β (Fig. 6I). Intriguingly, MI-increased pyroptosis-related proteins, such as cleaved IL-1β, cleaved-caspase 1, NLRP3, and N-terminal GSDMD, were reversed by AAV9-miR-654-3p-OE (Fig. 6J, K). These data demonstrated that overexpression of miR-654-3p in the heart rescued MI-induced cardiac dysfunction, attenuated myocardial fibrosis, and decreased pyroptosis.

### Overexpression of miR-654-3p altered the cardiac metabolism of MI mice

**Fig. 7:**
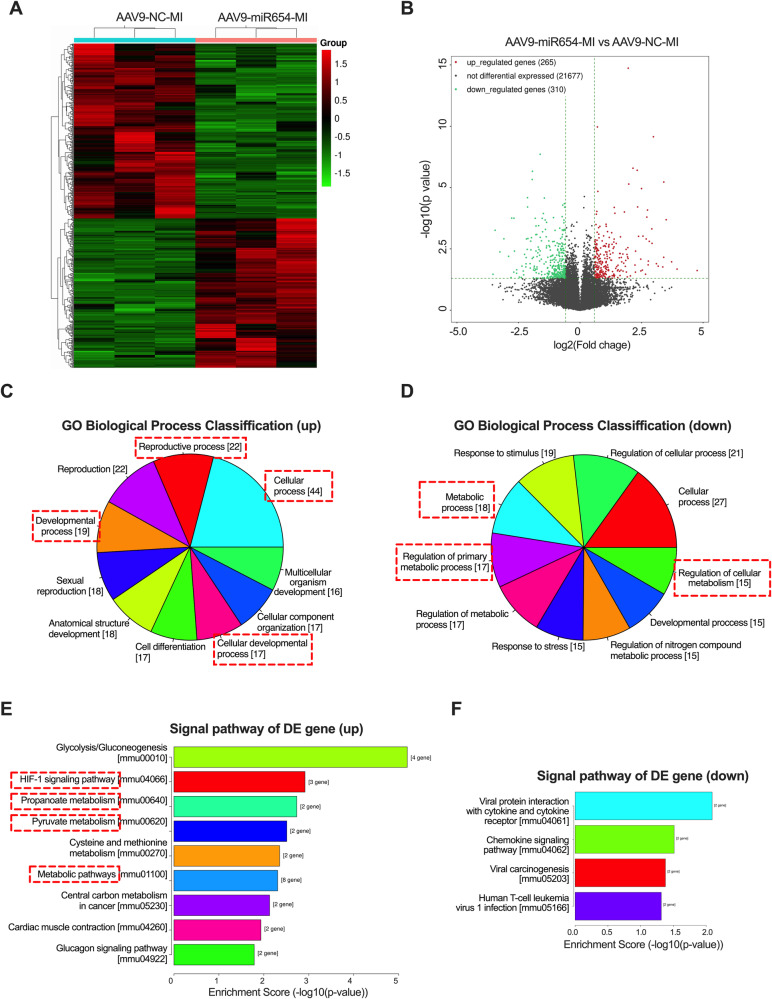
Metabolism is upregulated by overexpression of miR-654-3p in MI mice. **A** Heatmap of differentially expressed genes in AAV9-NC (*n* = 3) or AAV9-miR-654-3p-OE (*n* = 3)-treated MI mice hearts. **B** Volcano plot of different expressed genes between the two groups, fold change >2, *p*-value < 0.05. **C** GO biological process classification of upregulated genes in AAV9-miR-654-3p-OE vs. AAV9-NC heart as assessed by RNA-seq. **D** Gene ontology (GO) biological process classification of downregulated genes in AAV9-miR-3p-654-OE vs. AAV9-NC heart as assessed by RNA-seq. **E** Hierarchical clustering of the signal pathway of upregulated genes in AAV9-miR-654-3p-OE vs. AAV9-NC heart as assessed by RNA-seq. **F** Hierarchical clustering of the signal pathway of downregulated genes in AAV9-miR-654-3p-OE *vs*. AAV9-NC heart as assessed by RNA-seq. NC Negative control, OE overexpression, MI Myocardial infarction, GO Gene ontology.

To gain a complete understanding of the molecular mechanisms underlying miR-654-3p-mediated cardiac protection following MI, we conducted genome-wide RNA sequencing (RNA-seq) on heart tissues of AAV9-control- or AAV9-miR-654-3p-OE-infected MI mice 21 days post-MI. Hierarchical clustering analyses indicated that overexpression of miR-654-3p markedly altered the cardiac transcriptome following MI (Fig. 7A). In total, 488 genes (DEGs, adjust *p* value < 0.05) showed a difference between the AAV9-miR-654-3p-OE and AAV9-control group subjected to MI, 147 genes of which were upregulated, and 341 genes were downregulated (Fig. 7B). Gene ontology (GO) biological process analyses revealed that overexpression of miR-654-3p mainly upregulated genes related to the reproductive process, reproduction, cellular process, developmental process, multicellular organism development, cellular component organization, and cell differentiation (Fig. 7C). In addition, response to the regulation of the cellular process, metabolic process, and cellular metabolism are associated with the miR-654-3p-downregulated biological process (Fig. 7D). Similarly, Kyoto Encyclopedia of Genes and Genomes (KEGG) pathway analyses confirmed that upregulated genes are primarily related to metabolic-related pathways, glycolysis/gluconeogenesis, HIF-1 signaling pathway, propanoate metabolism, pyruvate metabolism cysteine, and methionine metabolism, and metabolic pathways (Fig. 7E). By contrast, downregulated genes are related to viral protein interaction with cytokine and cytokine receptors, chemokine signaling pathway, viral carcinogenesis, and human T-cell leukemia virus 1 infection (Fig. 7F). These data suggest that miR-654-3p overexpression improved heart function post-MI by mediating cardiac metabolic pathways.

### miR-654-3p overexpression promoted ATP production and mitochondrial respiration

**Fig. 8:**
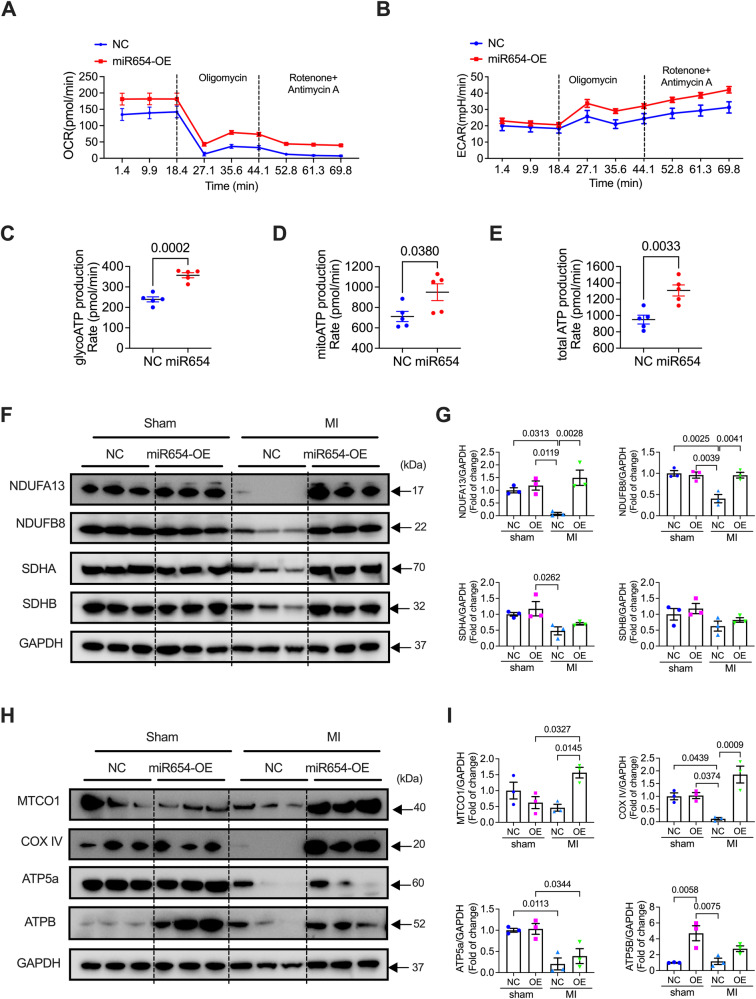
Overexpression of miR-654-3p promoted mitochondrial function. **A** oxygen consumption rate (OCR), **B** extracellular acidification rate (ECAR), and quantification of **C** glycolytic ATP production rate, **D** mitochondrial ATP production rate, and **E** total ATP production rate in NRCMs transfected with NC or miR-654-3p overexpression NRCMs (*n* = 5 in each group). **F** Representative western blotting images and **G** quantitative analysis of mitochondrial respiration-related proteins of complex I (NDUFA13 and NDUFB8) and II (SDHA and SDHB) in the heart of AAV9-NC- or AAV9-miR-654-3p-OE-infected mice subjected to sham or MI (*n* = 3 in each group). **H** Representative western blotting images and **I** quantitative analysis of mitochondrial respiration-related proteins of complex III (MTCO1), IV (COX IV), and V (ATP5a and ATPB) in the heart of AAV9-NC- or AAV9-miR-654-3p-OE-infected mice subjected to sham or MI (*n* = 3 in each group). Data were presented as mean ± standard error. Statistical significance was assessed by T-tests **(C**, **D**, and **E)** and one-way ANOVA (**G**, **I**). OCR oxygen consumption rate, ECAR extracellular acidification rate, ATP Adenosinetriphosphate, NC Negative control, NRCM Neonatal rat cardiomyocyte, OE overexpression, MI Myocardial infarction.

To determine the hypothesis that miR-654-3p overexpression (OE)-protected against MI was via the metabolic pathways, we performed the Seahorse Real-Time ATP Rate Assay in miR-654-3p overexpressed NRCMs. We found that mitochondrial respiration function measured by oxygen consumption rate (OCR) and extracellular acidification rate (ECAR) were markedly promoted by overexpression of miR-654-3p compared to the control group (Fig. 8A, B). We also tested the mitochondrial stress in miR-654-3p overexpression or knockdown NRCMs using Seahorse. The results showed that miR-654-3p overexpression weakly promoted mitochondrial stress, while miR-654-3p inhibition prevented the Mito-stress (Supplementary Fig. 4). The glycolytic ATP production rate was significantly promoted by the transfection of miR-654-3p (Fig. 8C). Simultaneously, mitochondrial and total ATP production was also increased (Fig. 8D, E). These in vitro data revealed that miR-654-3p overexpression promoted ATP metabolism. Next, we validated the in vivo role of overexpression of miR-654-3p on mitochondrial respiration function in MI mice. As shown in Fig. 8F–I, the decreased expression of complex I proteins (NDUFA13 and NDUF8) and complex IV (COX IV) in the hearts of MI mice were significantly rescued by miR-654-3p-OE. Although other complex proteins decreased in NC mice post-MI were rescued by miR-654-3p-OE, there were no significant differences (Fig. 8F–I). These data indicated that miR-654-3p-promoted ATP metabolism and mitochondrial function may be the fundamental mechanism of its protection against MI damage.

## Discussion

Here, we identified a novel and critical miRNA for regulating cardiac function called miR-654-3p, an essential mediator of cardiac metabolism and pyroptosis. We found that miR-654-3p is downregulated in response to myocardial infarction and is critical for cardiomyocyte pyroptosis, as depletion of miR-654-3p impairs cardiac morphology and function and promotes pyroptosis. Importantly, cardiac-specific overexpression of miR-654-3p protected the heart against MI damage by regulating metabolic signaling.

The functions of miRNAs, such as promoting the ability of proliferation, migration, and invasion in cells, are triggered mainly by the specific binding between miRNA and 3’-UTR of the target mRNA [26]. When they interact with each other, it may lead to the degradation of mRNA and a change in target protein levels [27]. Studies on miR-654 are less conducted. Lu et al. found that cell proliferation and metastasis were inhibited after miR-654-5p was transfected into oral squamous cell carcinoma cells [28]. miR-654-5p is highly expressed in breast cancer cells; overexpression inhibits growth and invasion and induces apoptosis in MDA-MB-468 and BT-549 cells [29]. miR-654-3p acted as an inhibitory role in nasopharyngeal carcinoma, liver cancer, thyroid cancer, ovarian cancer, colorectal cancer, and spinal osteosarcoma [21, 23, 27, 30–33]. miR-654-3p is lowly expressed in non-small-cell lung cancer tissue and inhibits tumor growth [34]. In cardiovascular-related diseases or cells, miR-654-3p participates in lncRNA TP73-AS1-regulated low-density lipoprotein-induced apoptosis of endothelial cells in atherosclerosis [35]. miR-654-3p is also participated in lncRNA ZDAS1-modulated cholesterol efflux in atherosclerosis [36]. Delivery of miR-654-5p via SonoVue microbubble ultrasound inhibits proliferation, migration, and invasion of vascular smooth muscle cells and arterial thrombosis and stenosis [37]. Our present data found that miR-654-3p is downregulated in response to MI and participated in the MI regulation.

The manifestations and mechanisms of various programmed cell death (PCDs), including apoptosis, necroptosis, ferroptosis, and pyroptosis, have been identified as associated with cardiovascular diseases [9, 12, 13]. Apoptosis is characterized by upregulated Bax, Caspase 3, and downregulated Bcl2 [9, 12]. Before this report, the current understanding of the molecular phenotype of the miR-654-3p in PCDs post-MI was still elusive. In our present data, although deficiency of miR-654-3p promoted cell programmed death, the levels of apoptotic markers, such as Bax, Caspase 3, and Bcl2, were not changed. Interestingly, miR-654-3p inhibition-induced cell death could be rescued by the pyroptosis inhibitor. Pyroptosis is a novel mechanism for regulating cell death and is an inflammation-mediated cell death due to the priming and activating of the inflammasome, which subsequently increases the permeability of the plasma membrane and releases inflammatory cytokines [18]. Inflammasome activation and pyroptotic cell death have been identified in various cardiovascular disease models, including acute MI, cardiac I/R, and HF [10, 14, 15, 38]. In our present research, we not only demonstrated that the deficient miR-654-3p-promoted PCD was suppressed by pyroptosis inhibitor VX765 but also found the miR-654-3p deficiency promoted the proptosis-related protein expression, such as NRLP3, cleaved Caspase 1, cleaved IL-1β, and GSDMD-N-terminal. Moreover, we manifested that deleting miR-654-3p aggravated MI-induced inflammation and promoted myocardial fibrosis progression, thereby exacerbating cardiac dysfunction, mainly regulated by promoting pyroptosis. Intriguingly, overexpression of miR-654-3p in the mice’s heart has a contrary effect on these functions, suppressing myocardial fibrosis and pyroptosis and rescuing cardiac function induced by MI.

Mitochondrial dynamics are activated to modify mitochondrial shape and structure to meet cardiomyocyte energy requirements [39]. The therapeutic strategies to attenuate MI-induced adverse cardiac remodeling include increasing the energy supply to the heart and switching the energy substrate preference to increase cardiac efficiency [40]. As mitochondrial dysfunction contributes to the pathogenesis of MI, research is focused on developing therapeutic strategies targeting mitochondrial homeostasis. Mitochondrial metabolism shifts from oxidative phosphorylation to glycolysis to reduce oxygen consumption and maintain ATP output in response to damage such as transient hypoxia or mild oxidative stress. In our results, cardiac overexpression of miR-654-3p significantly protected against MI-induced cardiac remodeling by decreasing myocardial fibrosis, inflammation infiltration, and pyroptotic activation. RNA sequencing showed that miR-654-3p overexpression promoted metabolic pathways. Further evidence showed that the overexpression of miR-654-3p promoted energy supply and mitochondrial respiration function.

However, the present study has many limitations, such as a large-scale trial of higher-profile mammalian models, such as minipigs or primates, is warranted further to translate this bench discovery into a treatment for MI. The secondary limitation is the target of miR-654-3p has yet to be identified. Another limitation is that the deep molecular pathway of miR-654-3p-moderated pyroptosis and mitochondrial function needs to be fully illustrated.

## Conclusion

The lines of evidence define a novelty pathogenic pathway involving the miR-654-3p-regulated pyroptosis and mitochondrial function responsible for MI. miR-654-3p may provide promising therapeutic effects to improve cardiomyocyte function in MI. Additionally, we found that promoting ATP production and mitochondrial function has a critical protection effect on MI.

## Methods

### Reagents and antibodies

Detailed information on reagents and antibodies can be obtained from Supplemental Materials and Methods 1.

### Deficiency or overexpression of miR-654-3p in mice heart

The methods of deficiency or overexpression of miR-654-3p in mice were presented in Supplemental Materials and Methods 2.

### Animal study and groups

Male 6-8-week-old C57BL/6J mice (wild-type, WT) were bought from GemPharmatech (Nanjing, China). All the mice were maintained in the Animal Center of Xiamen University at a specific pathogen-free (SPF)-grade laboratory. The mice were maintained on a 12-hour light/dark cycle.

To investigate the role of deficiency of miR-654-3p on cardiac function, 6-8-week-old male WT mice were injected with 100 µL AAV9-miR-654-3p sponge (HB-AAV9-cTNT-mmu-mir-654-3p-Null-LUC, 1.2 × 10^12^ vg/mL, Order number: HH20190125CJY-AAV01) (*n* = 9) or AAV9-control (HB-AAV9-cTNT-mmu-NC-Null-LUC, 1.1 × 10^12^ vg/mL, Order number: HH20190125CJY-AAV01-AAV00) (*n* = 9) via tail vein. At 3 and 6 weeks, echocardiography (ECG) detection was conducted to evaluate the heart function under anesthesia. After infection at 3 weeks, three mice in each group were euthanized by cervical dislocation under 3% isoflurane, and the heart tissues were collected to detect the Luciferase fluorescence and miR-654-3p expression using fluorescence microscopy and qRT-PCR. After ECG at 6 weeks, mice were euthanized. Then, the heart tissues and blood samples were collected for further detection.

To explore miR-654-3p deletion on MI-induced cardiac injury, mice were injected with 100 µL AAV9- miR-654-3p sponge or AAV9-control via tail vein. Three weeks after injection, mice were randomly divided into four groups: the AAV9-control sham group (*n* = 8), the AAV9-miR-654-3p sponge sham group (*n* = 10), the AAV9-control MI group (*n* = 12), and the AAV9-miR-654-3p sponge MI group (*n* = 12) and were subjected to MI or sham induction (Detailed methods of MI model is shown in Supplemental Materials and methods 3). The MI mice were subjected to ligate the anterior descending branch of the left coronary artery. The sham mice were threaded without ligation. ECG was performed to detect cardiac function 3 and 6 weeks after MI. In ECG detection and MI surgery, mice were anesthetized with isoflurane inhalation (Cat#: R510-22; RWD, Shenzhen, China) using a small animal anesthesia system (Cat#: R550, RWD, Shenzhen, China). After the last ECG detection, the mice were euthanized by cervical dislocation, and heart tissues and blood samples were collected. Part of the heart samples was embedded in a Tissue-Tek optimal cutting temperature (OCT) compound (Sakura, Tokyo, Japan) and stored at -80 °C for pathological staining and immunohistochemistry. The remaining hearts were stored at −80 °C for western blot assay.

To determine the effect of miR-654-3p overexpression on MI, mice were treated with 100 µL AAV9-miR-654-3p-OE (HB-AAV9-cTNT-mmu-mir-654-Null-LUC, 1.3 × 10^12^ vg/mL, HH20200720FJCJY-AAV01) and the AAV9-control (HB-AAV9-cTNT-mmu-NC-Null-LUC, 1.3 × 10^12^ vg/mL, HH2020720FJCJY-AAV00) via tail vein. Three weeks after injection, mice were randomly divided into four groups: the AAV9-control sham group (*n* = 9), the AAV9-miR-654-3p-OE sham group (*n* = 9), the AAV9-control MI group (*n* = 11), and the AAV9-miR-654-3p-OE MI group (*n* = 10) and were induced with MI or sham surgery (Detailed methods of MI model is shown in Supplemental Materials and methods 3). ECG detection was conducted to examine cardiac function at −21, 0, 1, 7, 14, and 21 days after surgery. After the last ECG, the mice were euthanized, and heart tissues and blood were collected. Part of the Heart samples was embedded with the Tissue-Tek OCT compound, and others were stored at −80 °C for further detection.

### RNA-fluorescence in situ hybridization (RNA-FISH)

The heart tissues from sham and MI mice (MI for 3 weeks after ECG) were embedded in the Tissue-Tek OCT compound (Sakura, Tokyo, Japan) and stored at −80 °C. Tissues were cut with 5 μm. The RNA FISH-Biotin Kit was applied to detect the miR-654-3p expression in the heart tissues. The instructions were as follows: rehydrated in citrate buffer, digested with proteinase K, denatured at 78 °C, incubated in the probe working solution for hybridization, and rinsed with a post-hybridization aqueous solution. The nuclei were stained with 4’,6-diamidino-2-phenylindole (DAPI). The pictures were captured using a fluorescence microscope (Leica, Germany). The intensity of miR-654-3p positive fluorecence in the hearts was statistically analyzed using Image J software (National Institutes of Health, MD, USA).

### Real-time qPCR

The heart tissues of the mice were homogenized with 1 mL TRIzol reagent (Cat#: 15596018; Life Technology, Carlsbad, CA, USA). Chloroform was added, mixed, and centrifuged. The upper layer was collected and combined with ethanol. After centrifuging, total RNA was collected and dissolved in DEPC water. The RNA concentration was measured and converted into cDNA using a reverse transcription kit (All-in-One™ miRNA First-Strand cDNA Synthesis Kit; GeneCopoeia, MD, USA). miR-654-3p expression was measured using a qPCR kit and mature miR-654-3p qPCR primers (Cat#: MmiRQP0764F, GeneCopoeia, MD, USA). SnRNA U6 qPCR primers were applied as the internal reference (Cat#: MmiRQP9002, GeneCopoeia, MD, USA).

### ECG

In vivo, heart function was imaged in the left lateral decubitus position using a Vevo 2100 animal ultrasound imaging system (VisualSonics, Toronto, Canada) with a 30-MHz high-frequency transducer as previously described [41–43]. The animals were anesthetized by inhalation of isoflurane (induction, 3%; maintenance, 1.5%). Temperature and respiration were continuously monitored during the ECG detection. Two-dimensional images were recorded in parasternal long- and short-axis projections, with guided M-mode recordings at the midventricular level in both views. Left ventricular ejection fraction (LVEF) and left ventricular fraction shortening (LVFS) were calculated using VEVO computer algorithms for at least three continuous cardiac cycles. ECG was performed by an investigator who was blinded to the grouping.

### Histological staining

Heart samples were embedded in OCT and cut cross-sectionally into 5-μm thick sections. Hematoxylin and Eosin (H&E) staining was performed for histological examination. Sirius red staining kit (Cat#: ab245887; Abcam) was used to identify collagen fibers. For H&E staining, sections were fixed using 4% paraformaldehyde. After being stained with H&E, sections were dehydrated with an ethanol gradient. For Sirius red staining, sections were fixed and sequentially incubated in picrosirius red solution and 0.5% acetic acid solution. Then, the sections were dehydrated with an ethanol gradient and sealed with neutral gum. After staining, images were taken with a TissueFAXS Plus panoramic scanner (Tissue Gnostics, Vienna, Austria) and were analyzed of the infarct area and myocardial fibrosis area using Image J (NIH, Bethesda, MD, USA). For each heart, 3-5 different sections were taken. The infarcted area and myocardial fibrosis value were determined by the percentage of positive staining area in the whole left ventricle area in each heart section.

### Neonatal rat cardiomyocyte (NRCM) isolation and transfection

The hearts of newborn suckling rats were collected, cut, and stirred overnight at 4 °C. Then the samples were incubated with 1.5 mg/mL collagenase II for 30 min at 37 °C on a shaker at 100 g. After the fibroblasts and endothelial cells adhered to the wall, the upper layer of the myocardial cell suspension was spread onto collagen solution-treated culture plates. After the cardiomyocytes attached to the wall and started beating, the medium was changed with a prewarmed maintenance medium (78% DMEM, 17% M-199 medium, 4% horse serum, and 1% penicillin-streptomycin solution) and incubated at 37 °C. Twelve hours later, NRCMs were incubated in 6-well plates for 12 hours. Then, the cells were transfected with miR-654-3p inhibitor (final concentration 100 nM), inhibitor control (final concentration 100 nM), miR-654-3p mimics (final concentration 100 nM), or negative control (final concentration 100 nM) using Lipofectamine^TM^ RNAi (8 μL) in Opti-MEM medium (1 mL). The transfection medium was replaced with DMEM (10% FBS) 8 hours post-transfection. Cells were maintained for 60-72 hours for further assays.

### Cell viability

Cell viability was determined using an automated cell counter (Countess 3FL, Thermo Fisher, USA). Briefly, after transfection with the control inhibitor and miR-654-3p inhibitor for 48 hours, NRCMs were treated with programmed inhibitors, VX765, Necrostatin, Ferrostatin-1, Z-VAD-FMK, and 3-MA for an additional 24 hours. NRCMs were collected and detected by the cell counter. Data were shown as the relative of control.

### ELISA

Blood samples were collected from the inferior vena cava of mice of the different groups. Collected blood samples were left to stand for 30 minutes at room temperature, followed by centrifugation at 2000g for 30 minutes to isolate the serum. Myocardial damage was assessed by measuring the biochemical indices of heart injury using ELISA kits, including mouse NT-proBNP ELISA Kit (Cat#: E-EL-M0834c), mouse CK-MB ELISA Kit (Cat#: E-EL-M0355c), mouse TNNI3/cTn-I ELISA Kit (Cat#: E-EL-M1203c), and AST/GOT Activity Assay Kit (Cat#: E-BC-K236-M) following the manufacturer’s instructions. The inflammation-related cytokines in the serum were measured by mouse IL-1βELISA Kit (Cat#: E-EL-M0037c) and mouse IL-18 ELISA Kit (Cat#: E-EL-M0730c) following the manufacturer’s instructions. Data were acquired using a Varioskan™ LUX multimode microplate reader (Thermo Fisher, Carlsbad, CA, USA).

### Flow cytometry

To test the miR-654-3p inhibitor on cell death, cells were transfected with the control inhibitor and miR-654-3p inhibitor for 72 h. To determine the effect of PCD inhibitors on miR-654-3p deficiency, cells were transfected with the control inhibitor and miR-654-3p inhibitor for 48 hours and/or treated with VX765, Necrostatin, Ferrostatin-1, Z-VAD-FMK, and 3-MA for another 24 hours. Cells were collected and resuspended in a binding buffer within Annexin V-FITC dye for 30 min, followed by PI staining for 5 min at room temperature. FL1 channel represents Annexin V-FITC, while FL3 channel represents PI. Cells were measured by flow cytometry (Beckman Coulter, California, USA).

### Immunoblotting

Heart tissues or NRCMs were lysed in RIPA lysis buffer (Cat#: R0010; Solarbio) supplemented with phosphatase inhibitor (Cat#: P1260; Solarbio) and protease inhibitor (Cat#: P6730; Solarbio). The protein supernatant was collected by centrifugation at 13200 g for 30 min. Protein concentration was measured using a BCA kit (Cat#: PC0020; Solarbio). Thereafter, the samples were denatured in the loading buffer (1/5 volume; Solarbio) in a metal bath at 95 °C for 10 min. The samples were resolved by sodium dodecyl sulfate–polyacrylamide gel electrophoresis (SDS-PAGE) and transferred onto Polyvinylidene fluoride (PVDF) membranes. The membranes were blocked in 5% nonfat milk in TBS buffer with 0.1% Tween 20 for 1 h, incubated with the following primary antibodies: Bcl2, Bax, Caspase 3, IL-1β, cleaved IL-1β, Caspase 1, NLRP3, GSDMD, GSAMD-N, GSDMD-C, SDHB, ATPB, NDUFB8, COX IV, UQCRC2, ATP5A, NDUFA13/GRIM19, NDUFS1, MTCO1, SDHA, GAPDH, β-actin, at 4 °C overnight at dilute concentration range of 1:1000 to 1:2000. Then, the secondary antibodies were incubated for 1 h. Protein band images were obtained using a chemiluminescence imaging system under the developer, and the bands were statistically analyzed using ImageJ (National Institutes of Health, MD, USA).

### Seahorse XF cellular ATP Rate test

We used the Agilent Seahorse XF Real-Time ATP Rate Assay Kit (103592-100) to measure real-time ATP rates (Glycolytic ATP and Mitochondrial ATP) using an Agilent XFe 24 Seahorse analyzer (Agilent, CA, USA). Briefly, after control mimics and miR-654-3p mimics transfection, NRCMs were seeded in the XF24 cell culture microplates (3 × 10^4^ cells/well) with a confluent of 70%–80%. The Agilent Seahorse Assay Medium was prepared with the following ingredients: 10 mM D-glucose, 2 mM glutamine, and 1 mM sodium pyruvate. The wells were tested under an inverted microscope to ensure the cells adhered to the plates. The XFe24 Seahorse instrument was calibrated by inserting a drug cartridge attached to a utility plate using an XF calibrant solution. Once instrument calibration was completed, the Seahorse microplate was loaded onto the Seahorse XFe Analyzer. The XF Real-Time ATP Rate Assay was then performed. The results were calculated using Agilent Seahorse XFe 24 analyzer software. Data was extracted using Graphpad Prism 9.0.

### Statistical analysis

GraphPad Prism 9 statistical software (GraphPad Software Inc, San Diego, CA) was applied to analyze these data and produce the figures. Final counts are presented as mean ± standard error (SEM). Student’s t-test was used to evaluate the size of mice chosen for each treatment group. For normally distributed data with one experimental variable, statistical analyses were performed by parametric analysis: unpaired (2-tailed) Student t-test for 2 groups and 1-way ANOVA with the Tukey multiple-comparison test for >2 groups. For non-normally distributed data with one experimental variable, statistical analyses were performed by nonparametric analysis: Mann-Whitney U test (2-tailed) for 2 groups and Kruskal-Wallis tests with post hoc Dunn multiple comparison tests for >2 groups. The P values were automatically produced by the software. Differences for which P < 0.05 were considered significant.

### Supplementary information

Supplemental materials

Unedited blotting images

## Data Availability

All data supporting the findings of this study are available in the article and Supplemental Material files. All original data for this study can be obtained from the corresponding author.
