# Supplementary material for: Pyroptosis and mitochondrial function participated in miR-654-3p-protected against myocardial infarction
Source: Cell Death Dis. 2024 Jun 4;15(6):393. doi: 10.1038/s41419-024-06786-4 (PMC11150501; doi:10.1038/s41419-024-06786-4)
Supplement: Supplementary file 1 — Supplemental materials [file 41419_2024_6786_MOESM1_ESM.docx]

**Supplemental materials**

**Pyroptosis and mitochondrial function participated in miR-654-3p-protected against myocardial infarction**

**Running title:** miR-654-3p protects against MI

**Supplemental** **materials and methods**

*Reagents and Antibodies*

Lipofectamine^TM^ RNAiMAX (Cat#: 13778150) was purchased from Thermo Fisher Scientific (Carlsbad, CA, USA). HBBAAV2/9-cTNT-GFP (1.1 × 10^12^ vg/mL, order NO: HH20190125CJY-AAV00), HBAAV2/9-mmu-miR-654-3p sponge-GFP (1.2 × 10^12^ vg/mL, Order NO: HH20190125CJY-AAV01), HBAAV2/9-cTNT-mmu-miR-654-3p-overexpression-GFP (1.3 × 10^12^ vg/mL, HH20200720CJY-AAV01), and HB-AAV2/9-cTNT-NC-LUC overexpression NC (1.3 × 10^12^ vg/mL, HH20200720CJY-AAV00) were ordered from HANBIO (Shanghai, China). The modified hematoxylin and eosin (H&E) staining kit (Cat#: G1121) was purchased from Solarbio (Beijing, China). Seahorse XF Realtime ATP Rate Assay Kit (Cat#: 103592-100) was purchased from Agilent (Palo Alto, CA, USA), and picrosirius red stain kit (Connective Tissue Stain, Cat#: ab63325) was purchased from Abcam (Cambridge, UK). All-in-One™ miRNA First-Strand cDNA Synthesis kits (Cat#: QP114), quantitative polymerase chain reaction (qPCR) primers against mature miRNA mmu-miR-654-3p (Cat#: MmiRQP0764), and Mouse snRNA U6 qPCR primers (Cat#: MmiRQP9002) were bought from GeneCopoeia Inc. (Rockville, MD, USA). The ABScript III RT Master Mix for qPCR with gDNA remover (Cat#: RK20429) and 2X Universal SYBR Green Fast qPCR Mix (Cat#: RK21203) were obtained from ABClonal (Wuhan, China). MiRNA-related reagents, such as rno-miR-654-3p-inhibitor (5'- AAGGUGAUGGUCAGCAGACAUA -3'), inhibitor control (5'-CAGUACUUUUGUGUAGUACAA-3'), rno-miR-654-3p-mimics (5'- UAUGUCUGCUGACCAUCACCUUGGUGAUGGUCAGCAGACAUAUU-3'), negative control (5'- UUCUCCGAACGUGUCACGUTTACGUGACACGUUCGGAGAATT-3'), and miRNA-654-3p fluorescence in situ hybridization (FISH) SA-Biotin were ordered from GenePharma (Guangzhou, China). Propidium iodide (PI, Cat#: P1304MP) was bought from Thermo Fisher. The programmed cell death inhibitors, including VX765 (Cat#: S2228), Necrostatin (Cat#: S8037), Ferrostatin-1 (Cat#: S7243), and Z-VAD-FMK (Cat#: S7023), were obtained from Selleck Chemicals (Houston, TX, USA). 3-MA (Cat#: MB5063) was bought from Meilun Bio (Dalian, China). The ELISA kits, including Mouse NT-proBNP (N-terminal pro-Brain Natriuretic Peptide) ELISA Kit (Cat#: E-EL-M0834c), Mouse CK-MB (Creatine Kinase MB Isoenzyme) ELISA Kit (Cat#: E-EL-M0355c), Mouse TNNI3/cTn-I (Troponin I Type 3, Cardiac) ELISA Kit (Cat#: E-EL-M1203c), Mouse IL-1β (Interleukin 1 Beta) ELISA Kit (Cat#: E-EL-M0037c), Mouse IL-18 (Interleukin 18) ELISA Kit (Cat#: E-EL-M0730c), and the Aspartate Aminotransferase (AST/GOT) Activity Assay Kit (Cat#: E-BC-K236-M) were purchased from Elabscience (Houston, TX, USA).

The primary antibodies, including anti-Bax (Cat#: ab32503), anti-Bcl2 (Cat#: ab196495), anti-pro Caspase 3 (Cat#: ab32150), anti-Cleaved Caspase 3 (Cat#: ab32042), anti-NLRP3 (Cat#: ab263899), anti-GSDMD (Cat#: ab219800), anti-N-terminal GSDMD (Cat: ab215203), ant-SDHB (Cat#: ab175225), anti-ATPB (Cat#: ab289890), anti-NDUFB8 (Cat#: ab192878), anti-COX IV (Cat#: ab202554), anti-ATP5A (Cat#: ab176569), and GAPDH (Cat#: ab181602), were bought from Abcam (Cambridge, UK). Anti-pro IL-1β (Cat#: AF5103) and anti-pro Caspase-1 (Cat: AF5418) antibodies were purchased from Affinity Bioscience (Jiangsu, China). Anti-NDUFA13/GRIM19 (Cat#: A5412), anti-β-actin (Cat#: AC026), and anti-MTCO1 (Cat#: A17889) antibodies were obtained from ABClonal (Woburn, MA, USA). Anti-SDHA (Cat#: 11998), anti-Cleaved-IL-1β (Cat#: 83186), and anti-Cleaved Caspase 1 (Cat#: 4199) antibodies were purchased from Cell Signaling Technology (Boston, MA, USA).

*Deficiency or overexpression of miR-654-3p in mice heart*

To knockdown miR-654-3p in murine hearts, the inverse complementary sequence of miR-654-3p (5’-AAGGTGATGGTCAGCAGACATA-3’) was inserted into the HBAAV-cTNT-MCS-ZsGreen vector with the following sequence (5’-acagaattcAAGGTGATGGTCAGCAGACATAtatacAAGGTGATGGTCAGCAGACATAacatcAAGGTGATGGTCAGCAGACATAtcttcaAAGGTGATGGTCAGCAGACATAttttttaagcttaca-3’). The intent fragment was obtained using PCR with the following primers: AAV-mmu-miR-654-3p-sponge-Eco-F (5’-acagaattcAAGGTGATGGTCAGCAGACATAtatacAAGGTGATGGTCAGCAGACATAacatcAAGGTGATGGTC-3’) and AAV-mmu-miR-654-3p-sponge-Hind-R (5’-acaaagcttaaaaaaTATGTCTGCTGACCATCACCTTtgaagaTATGTCTGCTGACCATCACCTTgatgtTATG-3’). Then, we copied and inserted the intent fragment into the vector (pHBAAV-cTNT-MCS-zsGreen, Hanbio Co, Ltd, Shanghai, China). The vector was then transfected to the DH5α competent cells to amplify them. The vector without downstream reverse sequence was used as the negative control. After sequencing, the vectors, including pHBAAV-cTNT-MCS-ZsGreen-miR-654-3p-sponge and pHBAAV-CMV-2A-ZsGreen-negative control, were cloned into the recombinant AAV9 frame vector (pAAV-RC vector and pHelper vector). The AAV9 vectors were then amplified in HEK293 cells. The viral titer was measured and enriched to more than 1.0 × 10^12^ vg/mL (Hanbio Co, Ltd, Shanghai, China).

To construct miR-654-3p overexpression AAV9, the sequence of miR-654-3p (5’- CTCGGTAAGTGGGAAGATGGTAAGCTGCAGAACATGTGTGTTTCTCATGTCATATGTCTGCTGACCATCACCTTTGGGTCTCTG -3’) was inserted into the HBAAV-cTNT-LUCi vector. The intent fragment was obtained using PCR with the following primers: AAV-mmu-miR-654-3p -Eco-F (5’- ACAgaattcCTCGGTAAGTGGGAAGATGGTAAGCTGCAGAACATGTGTGTTTCTCATGTC-3’) and AAV-mmu-miR-654-3p -Hind-R (5’-ACAaagcttCAGAGACCCAAAGGTGATGGTCAGCAGACATATGACATGAGAAACACACATGTTC-3’). Then, we inserted it into the vector (pHBAAV-cTNT-LUCi, Hanbio Co, Ltd, Shanghai, China). After that, the vectors were transfected to the Stb13 competent cells, cloned, sequenced, and cloned into the recombinant AAV9 frame vector. The AAV9 vectors were then amplified in 293T cells and enriched (Hanbio Co, Ltd, Shanghai, China).

*MI model*

After anesthetizing with inhalation of 3% isoflurane (Cat#: R510-22-10; RWD Life Science Co., Ltd., Shenzhen, China), the chest cavity was opened at the third and fourth intercostal space, and the heart was squeezed. The anterior descending branch of the left coronary artery was quickly ligated. Air in the chest was gently extruded, and anesthesia was stopped after suturing the skin. The antibiotic was injected through intramuscular. The surgery was completed after the mice awakened.

**Supplementary figures and figure legends**


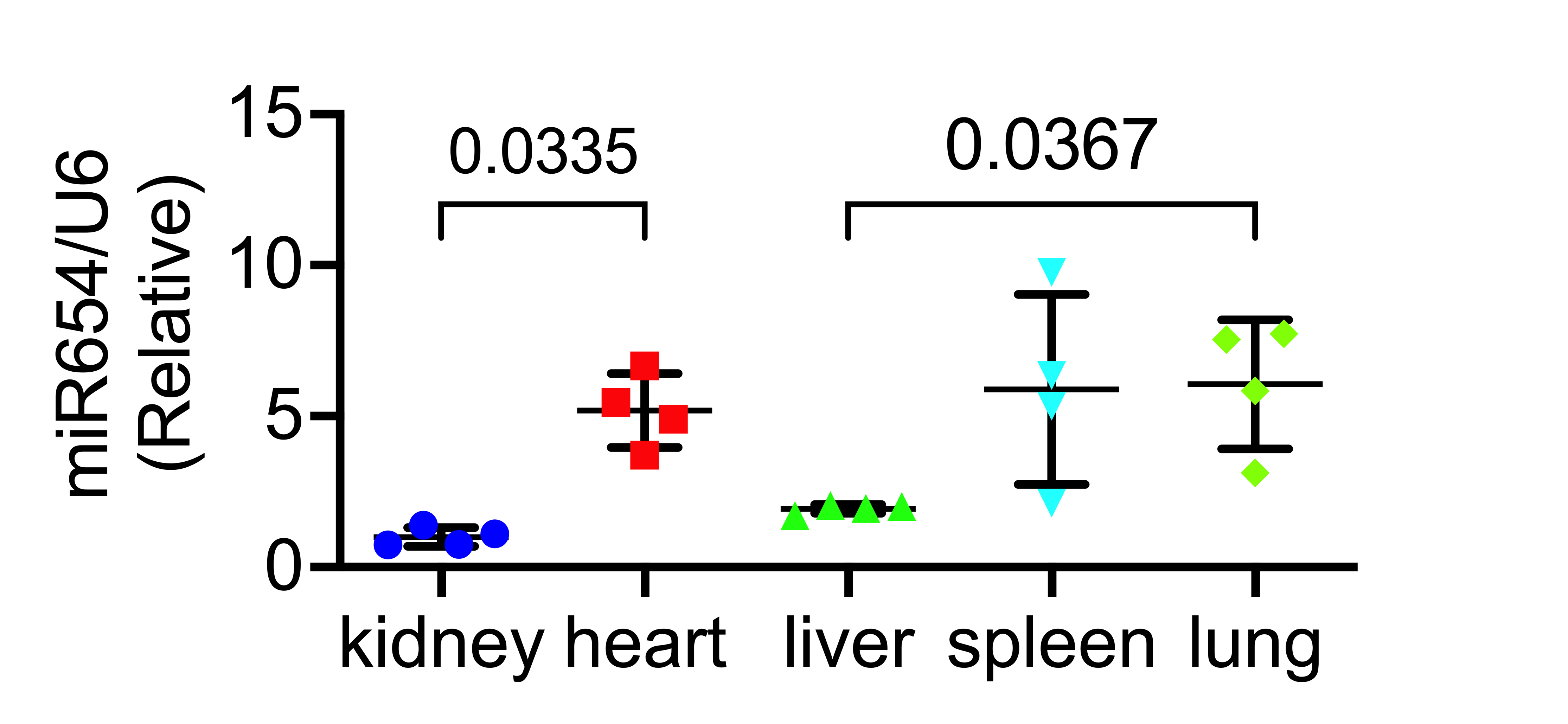


**Supplementary Figure 1. Distribution of miR-654-3p in deferent organs.** qRT-PCR analysis of miR-654-3p expression level in wild-type mice's kidney, heart, liver, spleen, and lung (n=4 in each group).


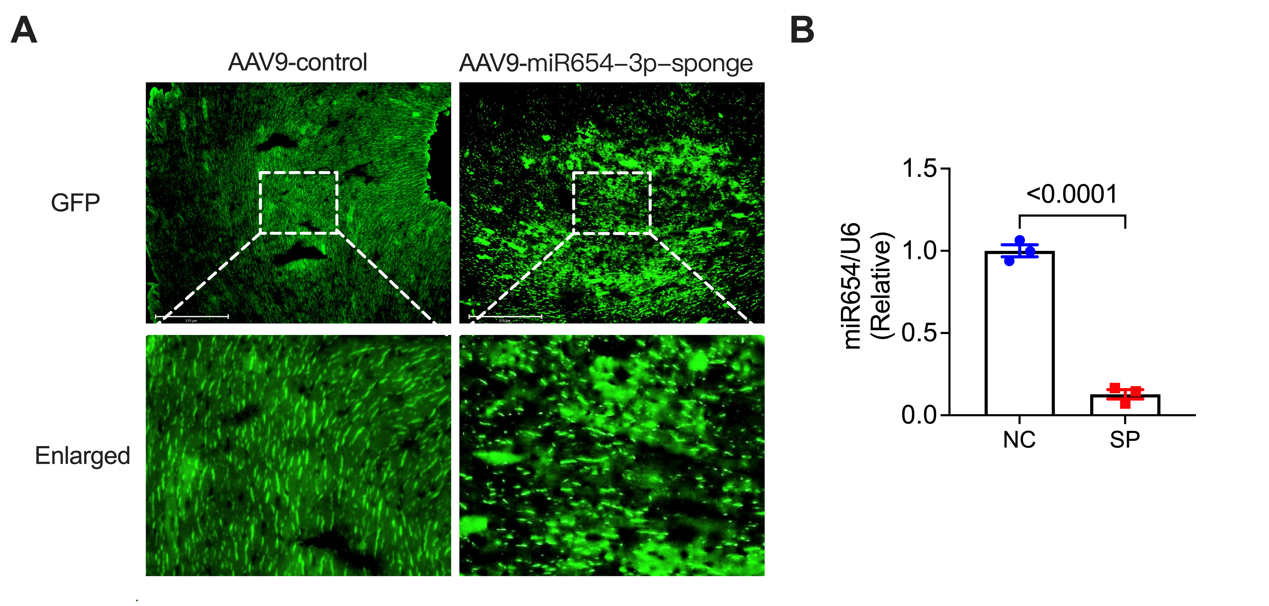


**Supplementary Figure 2. Identification of mice transfected with AAV9-control and AAV9-miR654-3p sponge. (A)** GFP expression in the heart of mice transfected with AAV9-control-cTNT-GFP and AAV9-miR654-3p sponge-cTNT-GFP. **(B)** qRT-PCR quantified the miR-654-3p expression in the heart transfected with AAV9-control-cTNT-GFP and AAV9-miR654-3p sponge-cTNT-GFP (n=4 mice in each group). Statistical analysis was performed using Student’s t-test for (**B)**.


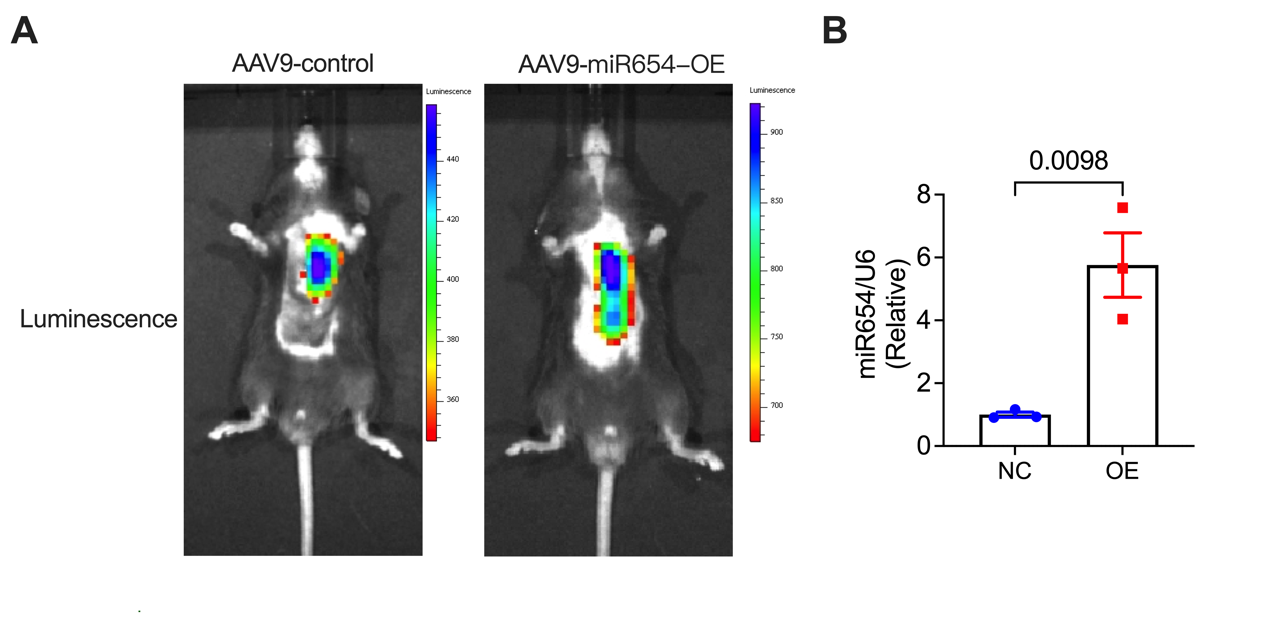


**Supplementary Figure 3. Identification of mice transfected with AAV9-control-LUC and AAV9-miR654-3p-overexpression-LUC. (A)** Luminescence in the heart of mice transfected with AAV9-control-cTNT-LUC and AAV9-miR654-3p overexpression-cTNT-LUC. **(B)** qRT-PCR quantified the miR-654-3p expression in the heart transfected with AV9-control-cTNT-LUC and AAV9-miR654-3p overexpression-cTNT-LUC (n=3 mice in each group). Statistical analysis was performed using Student’s t-test for (**B)**.

**
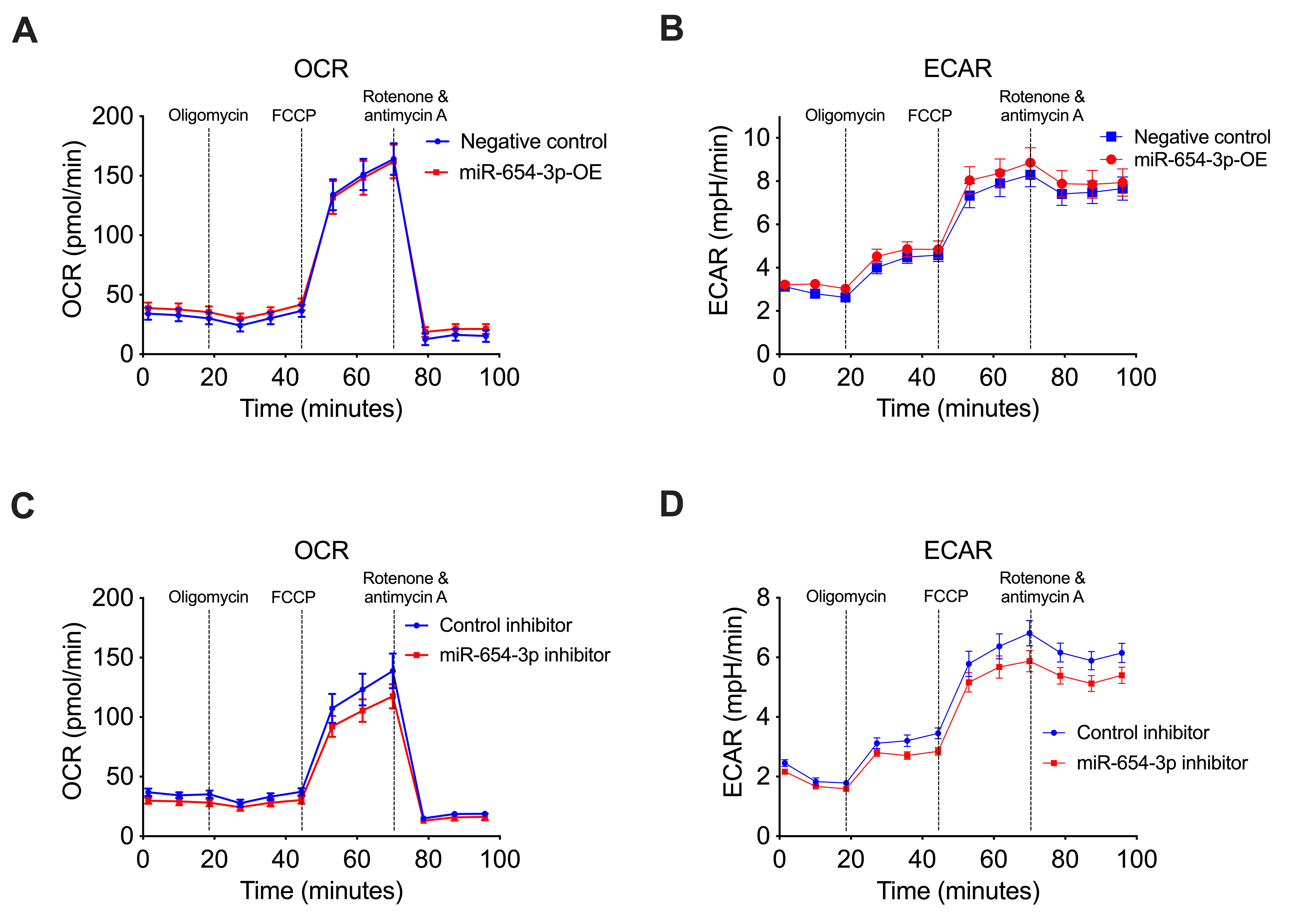
Supplemental Figure 4. The effect of miR-654-3p on mitochondrial stress.** **(A**) Seahorse detected oxygen consumption rate (OCR) and (**B**) extracellular acidification rate (ECAR) in NRCMs transfected with negative control (NC) or miR-654-3p overexpression NRCMs (n = 6 in each group). **(C**) Seahorse detected oxygen consumption rate (OCR) and (**D**) extracellular acidification rate (ECAR) in NRCMs transfected with Control inhibitor or miR-654-3p inhibitor NRCMs (n = 6 in each group).
