# Supplementary figures and images for: Pyroptosis and mitochondrial function participated in miR-654-3p-protected against myocardial infarction
Source: Cell Death Dis. 2024 Jun 4;15(6):393. doi: 10.1038/s41419-024-06786-4 (PMC11150501; doi:10.1038/s41419-024-06786-4)

**H**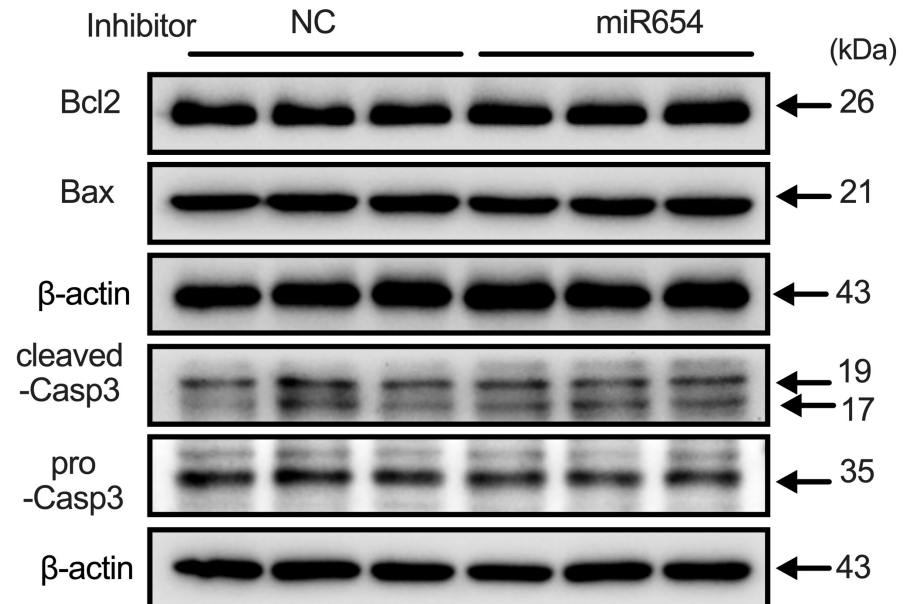

Figure 3H

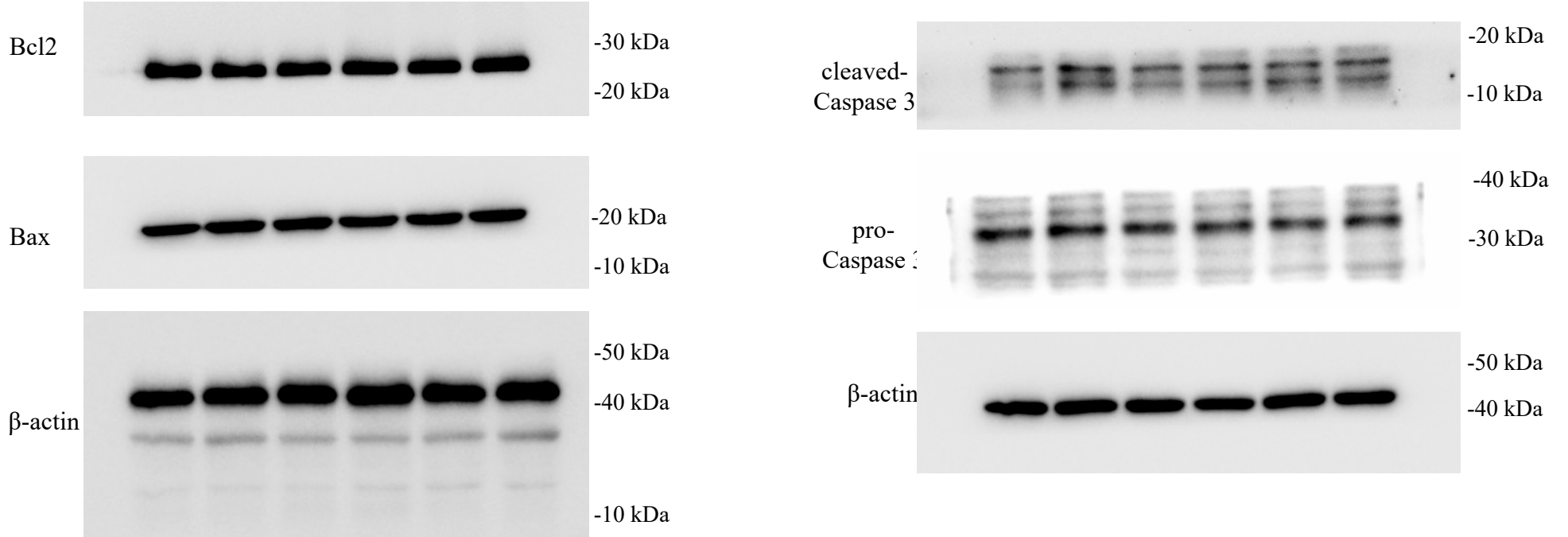

Figure 4E

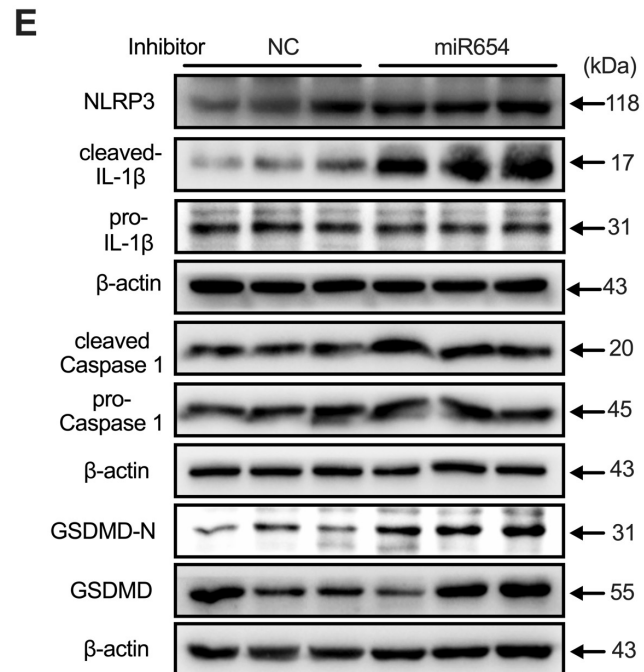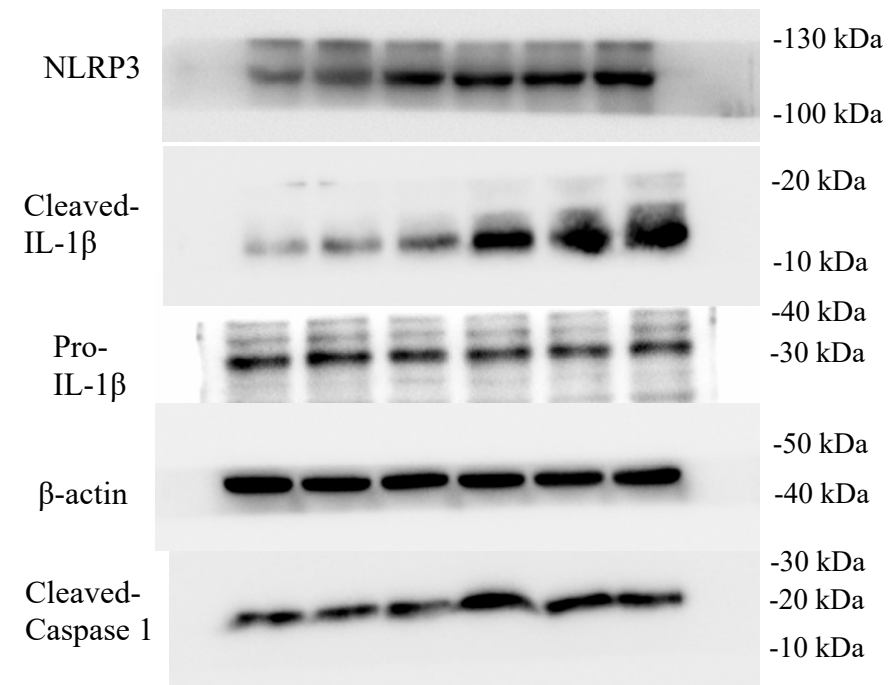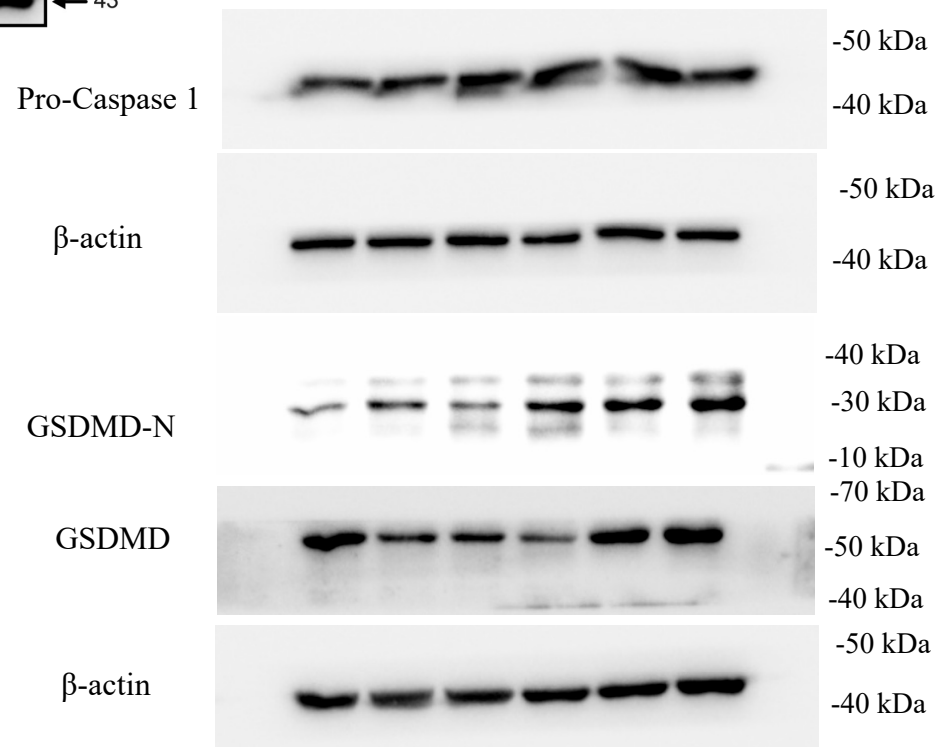

**K**

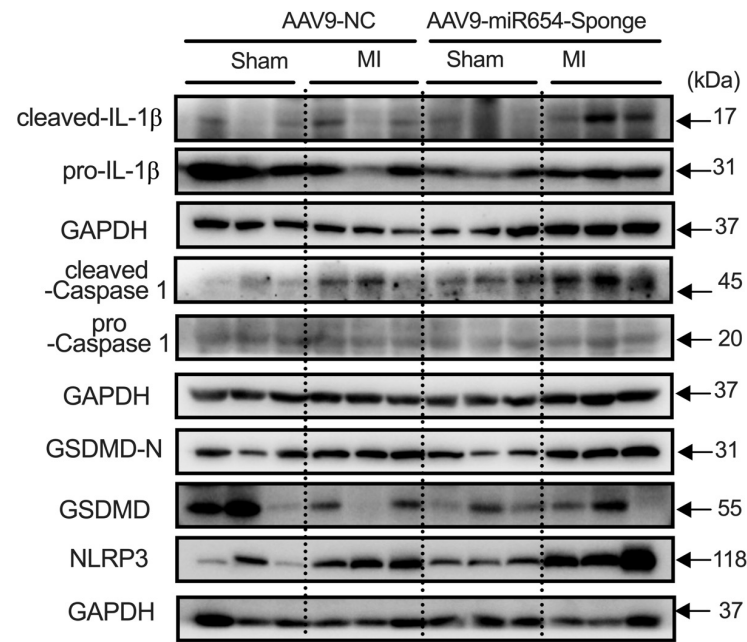

Figure 5K

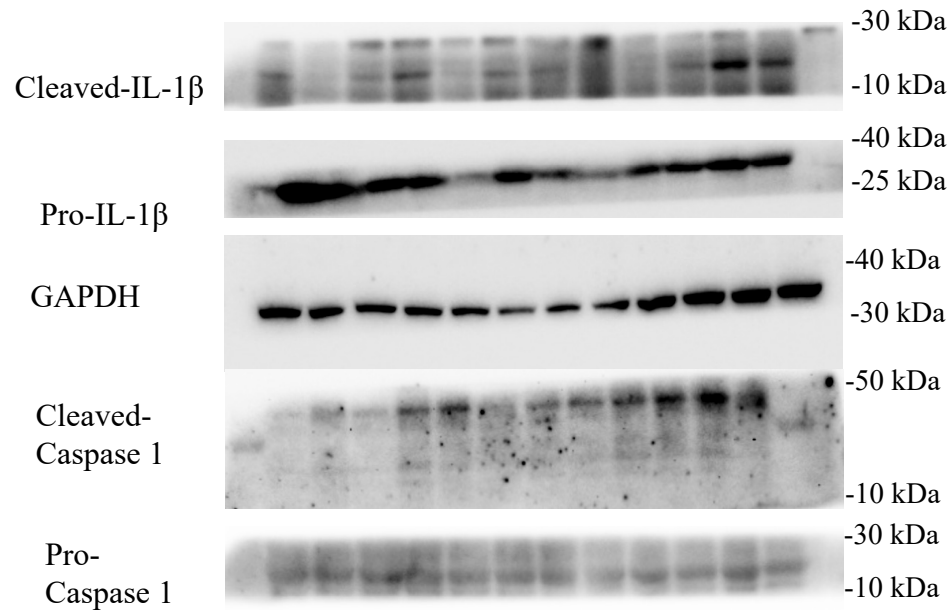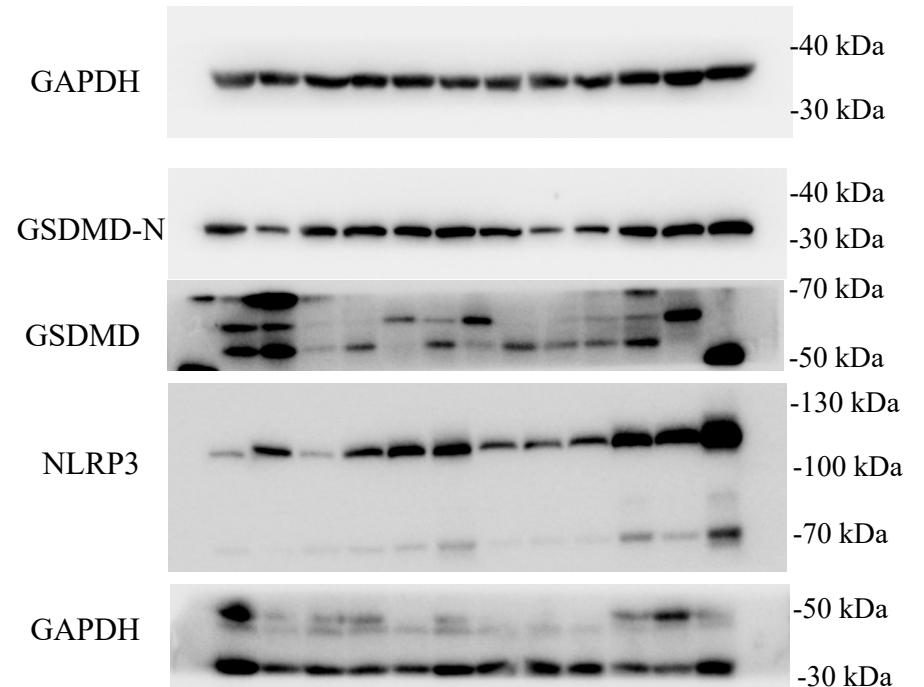

Figure 6J

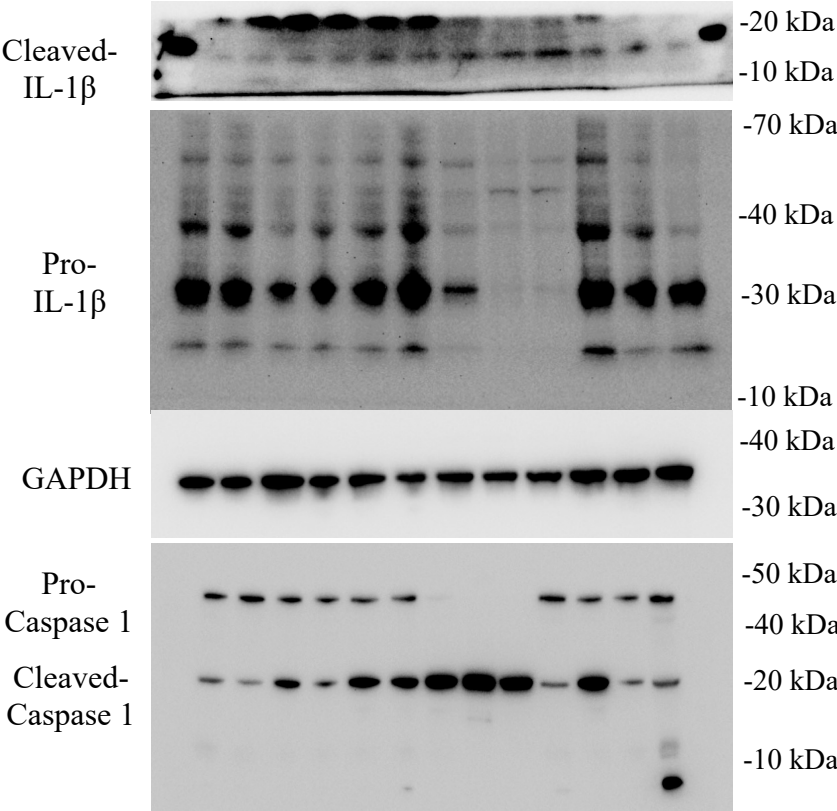

J

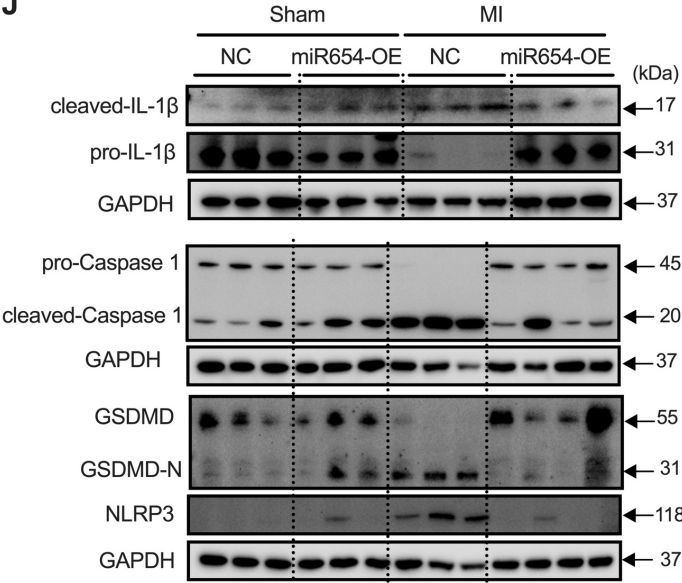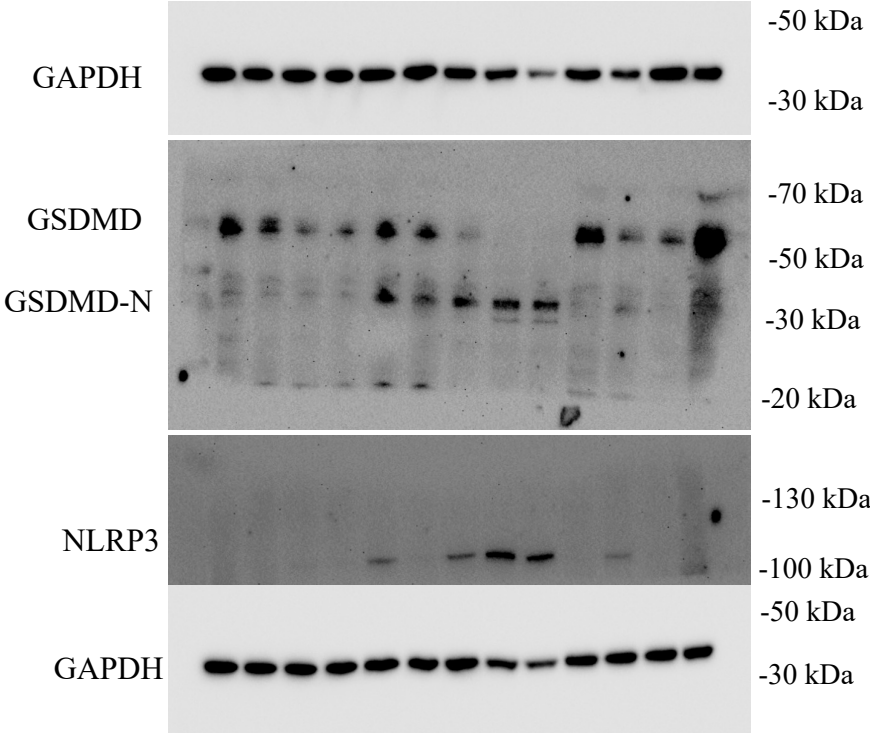

Figure 8F

F

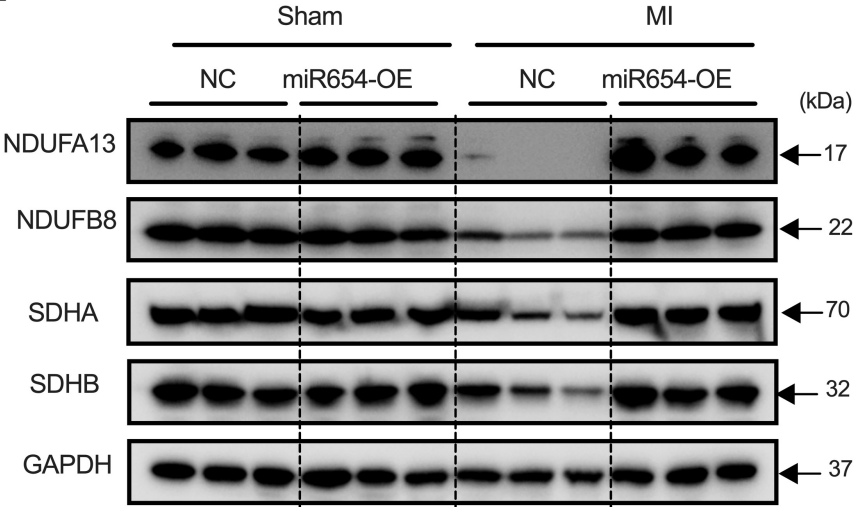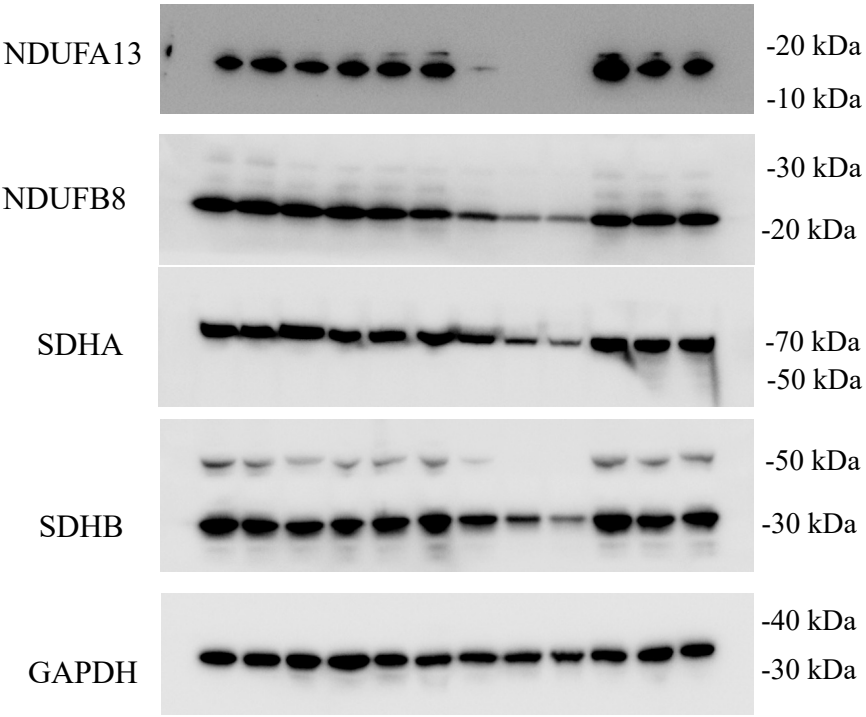

H

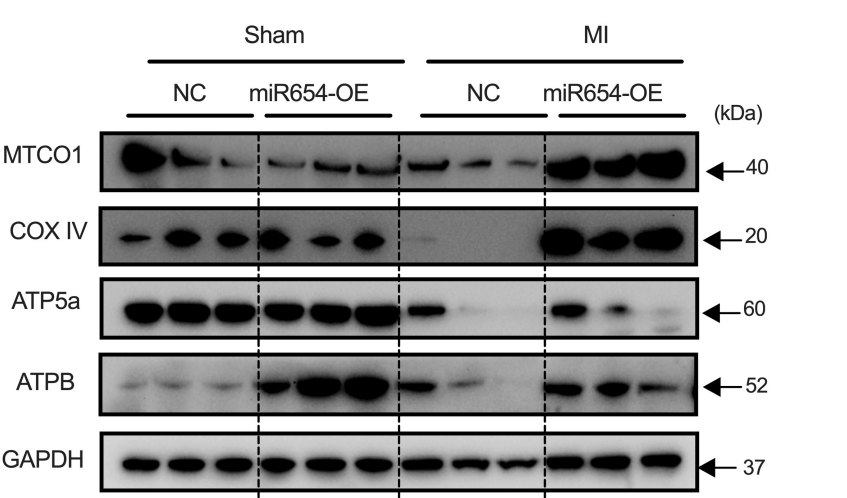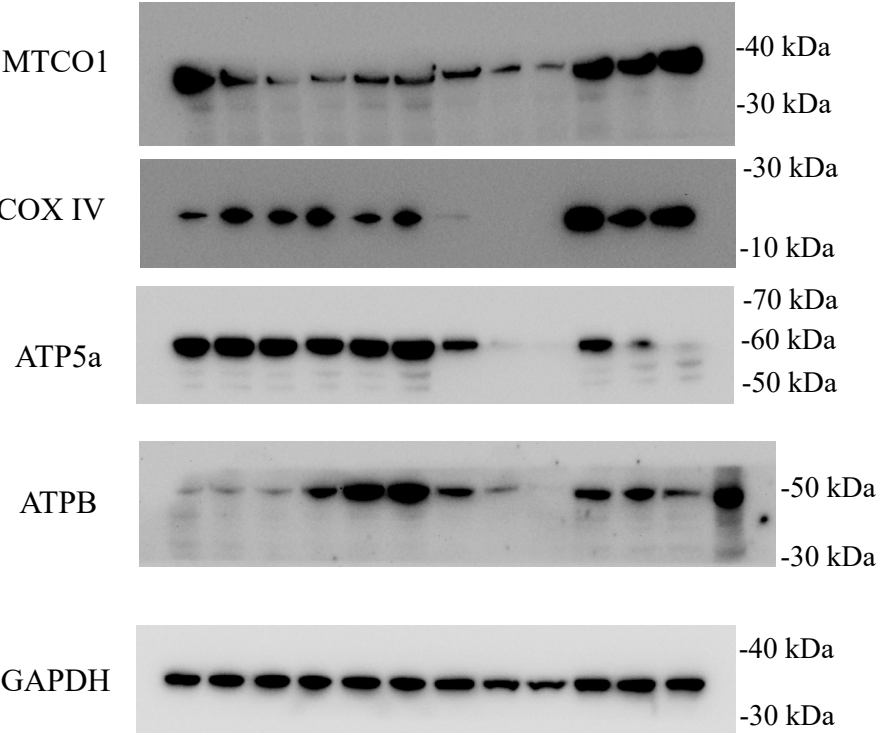

Supplement: Supplementary file 2 — Unedited blotting images [file 41419_2024_6786_MOESM2_ESM.pdf]
